# Harnessing Electrostatic Interactions for Enhanced Conductivity in Metal-Organic Frameworks

**DOI:** 10.34133/2021/9874273

**Published:** 2021-10-21

**Authors:** An-An Zhang, Xiyue Cheng, Xu He, Wei Liu, Shuiquan Deng, Rong Cao, Tian-Fu Liu

**Affiliations:** ^1^Department of Chemistry, School of Chemistry and Materials Science, University of Science and Technology of China, Hefei, Anhui 230026, China; ^2^State Key Laboratory of Structural Chemistry, Fujian Institute of Research on the Structure of Matter, Chinese Academy of Sciences, Fuzhou, Fujian 350002, China; ^3^University of the Chinese Academy of Sciences, Beijing 100049, China

## Abstract

The poor electrical conductivity of metal-organic frameworks (MOFs) has been a stumbling block for its applications in many important fields. Therefore, exploring a simple and effective strategy to regulate the conductivity of MOFs is highly desired. Herein, anionic guest molecules are incorporated inside the pores of a cationic MOF (PFC-8), which increases its conductivity by five orders of magnitude while maintaining the original porosity. In contrast, the same operation in an isoreticular neutral framework (PFC-9) does not bring such a significant change. Theoretical studies reveal that the guest molecules, stabilized inside pores through electrostatic interaction, play the role of electron donors as do in semiconductors, bringing in an analogous n-type semiconductor mechanism for electron conduction. Therefore, we demonstrate that harnessing electrostatic interaction provides a new way to regulate the conductivity of MOFs without necessarily altering the original porous structure. This strategy would greatly broaden MOFs' application potential in electronic and optoelectronic technologies.

## 1. Introduction

Exploration of electrically conductive metal-organic frameworks (MOFs) [1] offers exciting opportunities for fabricating electronic materials with the advantages of tunable structure, high crystallinity, and permanent porosity [2]. This would greatly expand the prospective applications of MOF materials [3–13]. Although increasing endeavors have been devoted to this field [14–17], MOFs with high conductivity are still rare, and the preparation of conductive MOFs has remained a big challenge to date. A common method to improve the conductivity of MOFs is to establish a pathway in a structure for the transportation of charge carriers [18], which can be achieved via deliberately choosing metal clusters and organic ligands with high charge-transport capability [19–21]. For example, the use of metal ions with partially occupied d orbitals (Fe, Cu) and ligands with heteroatoms (N, S) was found to increase the density of charge carriers and promote conductivity effectively [22–24]. However, this approach usually relies on tedious syntheses of specific organic linkers to obtain a new material. An alternative one is to choose exogenous species to infiltrate into the pores of a MOF to modify its poor conductivity. This method has the advantages of convenience and low cost. More strikingly, it allows for modulating the conductivity of a MOF without altering its basic framework structure. Although the feasibility of this method has been verified in several studies [25–29], the mechanism of the conductivity variation caused by the infilling of the guest molecules has yet to be well understood, which, however, is important, because it provides necessary information for designing and preparing new semiconductive MOFs with rich structural variations and modifiability.

In this work, we incorporated an anionic complex Zn-S·TA (Bis(tetrabutylammonium) Bis(1,3-dithiole-2-thione-4,5-dithiolato)zinc) into a cationic framework, by which, for the first time, electrostatic interaction was exploited to stabilize the guest molecules and modulate the conductivity of MOFs. Consequently, the conductivity increased dramatically by five orders of magnitude from 1.01 × 10^−9^ S·cm^−1^ to 1.06 × 10^−4^ S·cm^−1^, being an outstanding performance among all the reported electrically conductive MOFs. The additive amount in the structure was found to be extremely low; therefore, it presented an efficient strategy for regulating MOF conductivity without altering the pristine structure and porosity. Theoretical calculation suggests that the electrostatic interaction plays an important role in stabilizing the intercalated small molecules and brings in an analogous n-type semiconductor mechanism for electron conduction. We found that the infiltration of charge guest molecules into an ionic framework can dramatically increase the charge carrier concentration and the dispersion of the conduction bands of the framework. As a result, the weakly bound electrons on the donor purity levels are readily excited to the conduction bands, which leads to a dramatic improvement in conductivity.

## 2. Results

### 2.1. Zn-S@PFC-8 Fabrication and Characterization

**Scheme 1:**
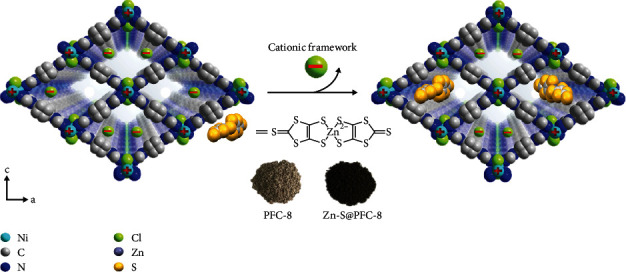
Schematic representation of loading Zn-S^2-^ ions into PFC-8.

**Figure 1:**
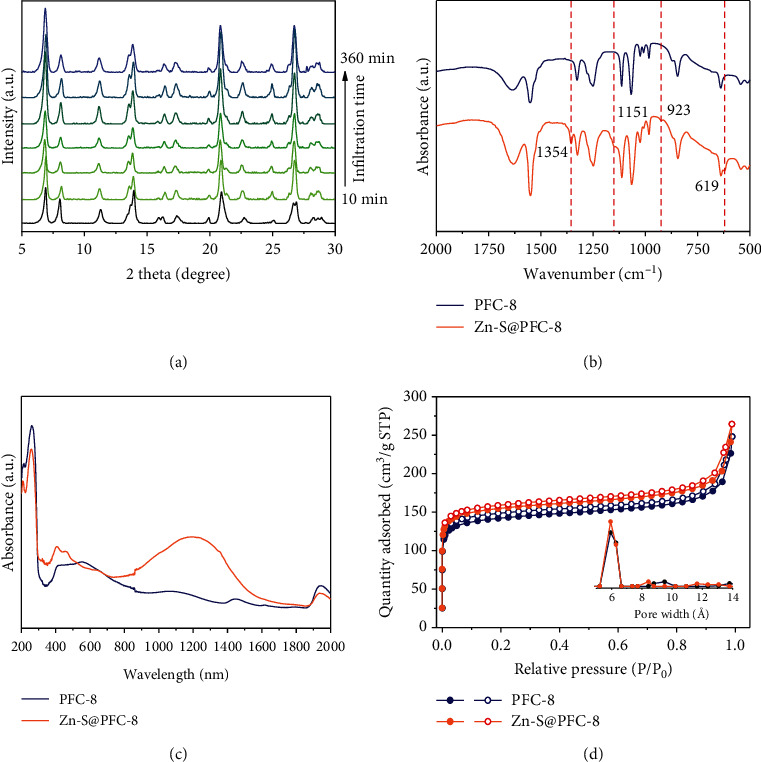
(a) Time-dependent PXRD patterns of PFC-8 upon Zn-S^2-^ infiltration. (b) IR spectra of PFC-8 and Zn-S@PFC-8. (c) UV-Vis-NIR spectra of PFC-8 and Zn-S@PFC-8. (d) N_2_ isotherm curves of PFC-8 and Zn-S@PFC-8. Inset: the pore size distribution of PFC-8 and Zn-S@PFC-8.

**Figure 2:**
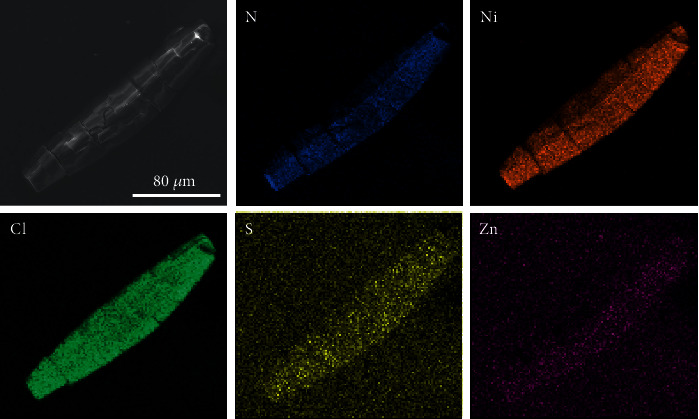
Energy Dispersive Spectroscopy (EDS) elemental mapping of Zn-S@PFC-8.

PFC-8 (Ni(BTAB)Cl_2_) is a MOF with a cationic backbone and isolated Cl^−^ as counterions inside pores [30]. Single-crystal X-ray diffraction analysis reveals that each Ni(II) ion is square-planar coordinated by four N atoms and axial-occupied by two bridging Cl^−^ to form a Ni(II) metal chain. BTAB ligands further connect adjacent metal chains to construct a three-dimensional network with a quadrilateral channel about 21 × 15 Å (Scheme 1). PFC-8 leaves a net positive charge on metal nodes and attracts Cl^−^ counterions in channels for charge balance. Zn-S·TA, composed of anionic complex and ammonium counterions [31–33], was chosen as an additive to replace Cl^−^ ions inside pores with Zn-S^2-^ ions through an ion-exchange method. The replacement was conducted by immersing PFC-8 in Zn-S·TA solution for 18 hours at room temperature (25°C) (see Figure S1). Ion chromatography was used to monitor the ingredient changes of supernatant during the ion-exchange process (Figure S2). In the first seven hours, the dramatic increase of Cl^−^ concentration in the supernatant indicated that the isolated Cl^−^ counterions inside MOF pores were partially replaced by Zn-S^2-^ ions. From the seventh hour to the ninth hour, the Cl^−^ concentration in the supernatant remained almost unchanged, indicative of achieving ion-exchange equilibrium, during which the color of the powdery sample changed from grey to dark brown. The Zn-S^2-^ content (chemical formula: ZnS_10_C_6_) in PFC-8 was determined to be 0.9 wt% (0.7 mol%) based on AA (Atomic Absorption) spectroscopy results (Table S1), equivalent to accommodating 0.03 Zn-S^2-^ molecules in each unit cell. PFC-8 even maintained the single-crystal form after loading and was capable of single-crystal X-ray determination (Figure 1(a)). The crystallographic result shows that Zn-S@PFC-8 keeps the same structure as pristine PFC-8 (Table S2). However, the exact position of Zn-S^2-^ inside pores cannot be determined because of the low concentration and disordered distribution. The infrared (IR) spectrum of Zn-S@PFC-8 shows four new peaks at 1354 cm^−1^, 1151 cm^−1^, 923 cm^−1^, and 619 cm^−1^. Since the fingerprint area of IR (around 650-1300 cm^−1^) usually reflects the structure and energy changes of the sample, these new peaks suggest that there may exist some interactions between Zn-S^2-^ and the framework (Figure 1(b)). Since the content of guest molecules is low, there is no significant change in nitrogen uptake after Zn-S^2-^ loading (Figure 1(d)). Moreover, the incorporation of Zn-S^2-^ molecules in PFC-8 gave rise to new broad adsorption on the diffuse reflectance spectrum (DRS) in the range of 1000-1400 nm, which is absent for PFC-8 and Zn-S·TA (Figure 1(c) and Figure S3). The different adsorption spectra indicate the possible change in the electronic structure of these materials. Energy Dispersive Spectroscopy (EDS) elemental mapping images indicate the even distribution of S and Zn elements on particles (Figure 2 and Table S3). In order to further illustrate the inclusion of Zn-S^2-^ inside PFC-8, Zn-S@PFC-8 was etched with the thickness of 5 nm, 10 nm, 25 nm, and 50 nm, respectively, prior to X-ray photoelectron spectroscopy (XPS) analysis. As shown in Figure S4, the intensity of the S element keeps consistent with different etching depths. Together with EDS mapping and AA analysis, we can draw a safe conclusion that Zn-S^2-^ was successfully encapsulated inside the pores of PFC-8.

### 2.2. Electrical Conductivity Measurements of PFC-8 and Zn-S@PFC-8

**Figure 3:**
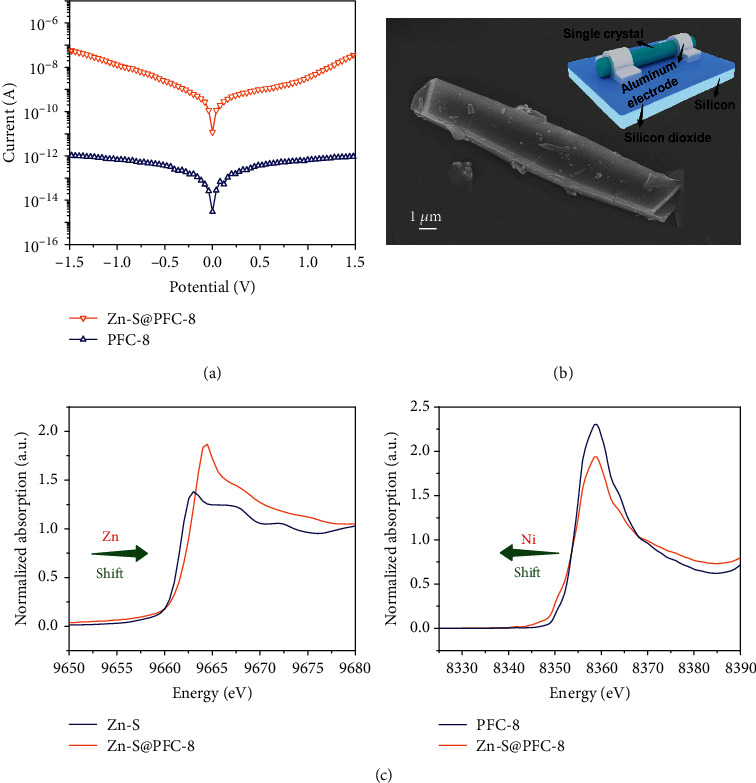
(a) *I*–*V* characteristics of PFC-8 single crystal before and after Zn-S^2-^ loading at 25°C. (b) SEM image of Zn-S@PFC-8. The inset shows the schematic diagram of the two-probe method. (c) The normalized XANES spectra at Zn and Ni K-edge of PFC-8 and Zn-S@PFC-8.

Thanks to the large crystal size, electrical conductivities in this work can be measured using a single crystal by a two-point probe for more accurate results (Figure 3(b)). The as-synthesized PFC-8 show moderate conductivity among reported MOF materials (1.01 × 10^−9^ S·cm^−1^). In contrast, the conductivity of Zn-S@PFC-8 (1.06 × 10^−4^ S·cm^−1^) is about five orders of magnitude greater than that of pristine PFC-8 confirmed by repeated measurements (Figure 3(a), Figures S5 and S6, and Table S4). The striking difference in conductivity inspired us to further look into the conductive mechanism. As shown in X-ray absorption near-edge structure (XANES) spectra, the Zn K-edge shifts to higher energy after Zn-S^2-^ loading. Moreover, it can be observed that the white line intensity of Zn-S@PFC-8 is much higher than that of PFC-8 for the Zn K-edge (Figure 3(c), left panel) but an opposite trend for the Ni K-edge. These results indicate that the incorporation of Zn-S^2-^ causes an increasing oxidation state for Zn and a diminishing oxidation state for Ni [34, 35]. It is interesting to note that, although there is an obvious change being observed in XANES, both PFC-8 and Zn-S@PFC-8 show very similar results on extended X-ray absorption fine-structure spectroscopy (EXAFS). The EXAFS equation gave rise to very close *N* (number of neighboring atoms) and *R* (distance to the neighboring atom) values for these two materials (Figures S7 and S8 and Table S2). As we know, EXAFS is sensitive to near-neighbor coordination shells. Therefore, these results indicate that loading Zn-S^2-^ in PFC-8 did not alter the framework as well as the Ni coordination sphere but dramatically changed the electron density of Ni center [36]. This phenomenon can be well explained by the electrostatic interaction existing between the framework and the Zn-S^2-^, which does not disturb the coordination environment but pulls the electron cloud close to the Ni nucleus.

### 2.3. Effects of Electrostatic Interaction on the Electrical Conductivity

**Scheme 2:**
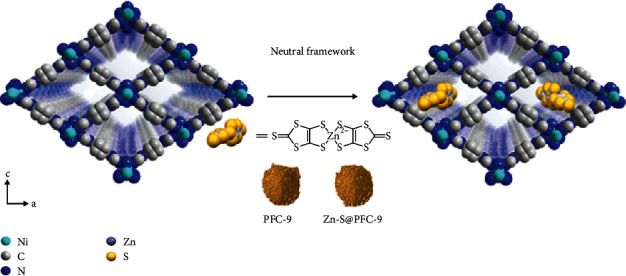
Schematic representation of loading Zn-S^2-^ ions into PFC-9.

**Figure 4:**
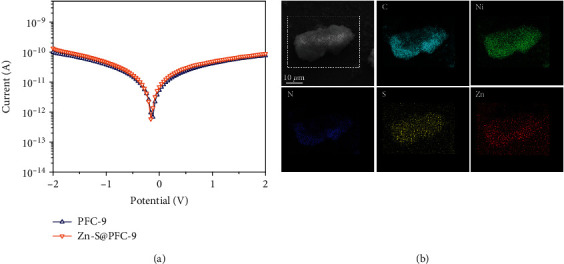
(a) *I*‐*V* characteristics of PFC-9 and Zn-S@PFC-9. (b) EDS elemental mapping of Zn-S@PFC-9.

The increase of conductivity in cationic PFC-8 after infiltrating ionic guest molecules is much noticeable than other reported studies using neutral MOFs (Table S10). Taking this cue, we speculate that the electrostatic interaction plays a vital role in this drastic improvement. Therefore, a neutral framework PFC-9 (Ni(H_2_DPB)) [30], possessing similar ligands, identical metal centers, and same topology with PFC-8, was prepared and followed with the same Zn-S^2-^ loading procedure (denoted as Zn-S@PFC-9) for electrical conductivity investigation (Scheme 2 and Figure S9). Since single crystals were not available for PFC-9, the conductivity measurements were performed on pressed square shape pellets using a two-point probe setup. In sharp contrast, the conductivity of Zn-S@PFC-9 pellets did not show a noticeable change with infiltrating Zn-S^2-^ inside the pore of the structure (Figures 4(a) and 4(b), Tables S5 and S6, and Figures S10 and S11).

Moreover, a dramatic difference in electrochemical property can also be observed from the CV scan. As shown in Figure S12, both PFC-8 and PFC-9 show a pair of reversible redox peaks around 0.62 V and 0.85 V, which can be ascribed to the redox reaction of Ni^2+^/Ni^3+^ or Ni^2+^/Ni^4+^ [37–40]. These peaks remained after loading Zn-S^2-^ in PFC-9, indicating that the guest molecules did not change the redox potential of Ni centers. However, these peaks disappeared in the CV scan of Zn-S@PFC-8, which is a strong indication of the change in electron delocalization. This phenomenon is usually observed in some metal complexes where the variation of the coordination environment can greatly change the electron delocalization of metal clusters and result in different electrochemical properties [41, 42]. Therefore, by loading the same guest molecules in a similar structure but different charge nature, we can infer that the electrostatic interaction between guest molecules and the host framework (including both ligands and metal centers) plays a vital role in altering the electrochemical properties of materials.

### 2.4. Theoretical Calculation for Zn-S@PFC-8

**Figure 5:**
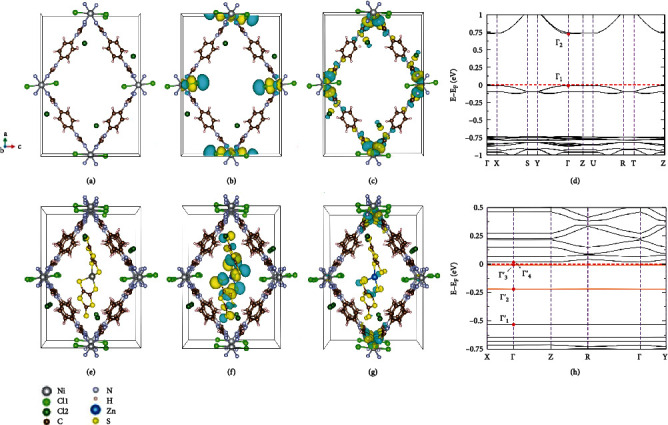
Optimized crystal structure model, calculated orbitals for VBM and CBM, and band structures near EF for (a–d) *P*2_1_2_1_2_1_-PFC-8 and (e–h) a-mod1 of Zn-S@PFC-8, respectively.

To understand the mechanism of the conductivity change, theoretical calculations based on density functional theory (DFT) were performed by using the Vienna Ab initio Simulation Package (VASP) [43]. Considering the strong electron correlation effects of the d electrons of the Ni atoms, we used the DFT+U scheme [44] in Dudarev's approach [45] with an effective Coulomb parameter *U*_eff_ = 2.5 eV. Taking into account the measured paramagnetic property of the material (Figure S13), a paramagnetic ground state has been used to perform all electronic calculations throughout this work. A structure model of PFC-8 with the space group *P*2_1_2_1_2_1_ (Figure 5(a)) was found through energy optimization and space group screening (Tables S7 and S8 and Figure S14).

As the length of Zn-S^2-^ molecule (~14.234 Å) is larger than twice of the *b*-axis (~7.032 Å) of PFC-8, a supercell with *a*′ = *a*, *b*′ = 3*b*, and *c*′ = *c* (*a*, *b*, and *c* represent the basis vectors of the unit cell of the *P*2_1_2_1_2_1_-PFC-8 model) was built for the calculations. Considering the structural symmetry, size, orientation, and experimental concentration of Zn-S^2-^ molecules in the Zn-S@PFC-8, seven different structure models with the same chemical formula of C_10_H_8_N_6_NiCl_1.83_(ZnS_10_C_6_)_0.083_ have been built by putting one Zn-S^2-^ molecule into the PFC-8 supercell and removing two Cl^−^ ions for charge balance (Figure S15, 339 atoms within each cell). Note that the Zn-S^2-^concentration in our theoretical study is higher than the experimental AA and EDS results in order to save calculation time and expense. However, this structure model is reasonable considering that the shortest distances between two nearby Zn-S^2-^ molecules are larger than 6.89 Å with negligible intermolecular interaction. Through investigating the displacements of atoms from their initial locations in the starting model to those in the optimized models (Figure S16), we found that the Zn-S^2-^ molecules tend to stay at the center of the channel. This result indicates that electrostatic interaction together with the steric hindrance plays an important role in stabilizing the Zn-S^2-^ molecules inside the pores. Although the Zn-S^2-^ molecules in the different models were found to locate at different positions, the calculated total energies of these models are only marginally different (<1 meV/atom, see Table S9), which indicates that the Zn-S^2-^ molecules may locate or orient randomly in the pores of PFC-8 and show significant dynamics. This result is consistent with the single-crystal X-ray measurements which revealed a disordered distribution of the infiltrated Zn-S^2-^ molecules.

The band structure and density of states (DOS) of *P*2_1_2_1_2_1_-PFC-8 (Figure 5(d) and Figure S17) show a bandgap (*E*_*g*_) of 0.737 eV, which can be smaller than the actual value due to the well-known deficiency of the DFT calculation. As shown in Figures 5(b) and 5(c), the state of the valence band maximum (VBM), Γ_1_, consists mainly of the Ni-Cl1 (Cl atom from the backbone) d-p orbitals with antibonding interaction, while the state of the conduction band minimum (CBM), Γ_2_, is mainly made up of Ni-N d-p orbitals of antibonding interaction (for atom labels and other details, see Table S7 and Figure S17). Besides, the overlaps between the Ni and Cl2 (Cl^−^ counterion) orbitals are negligible, whereof the nonbonding Cl2-3p orbitals lie around -0.8 eV (Figure S17). These results indicate that the interactions between Ni and Cl2^−^ are of dominatingly ionic nature and thus are much weaker than the covalent interactions.

Among the seven structure models with Zn-S^2-^ molecules infiltrating into the PFC-8, five of our structure models (a-mod1, c-mod1, b-mod3, b-mod4, and b-mod5) give rise to substantially smaller band gap values, *E*_*g*_, around 0.018 eV (Table S9) compared to that, ~0.74 eV, of PFC-8. For the rest two models (b-mod1 and b-mod2), the calculations result in Fermi levels crossing some bands indicating possible metallic states, which is, however, incompatible with the experimental conductivity of Zn-S@PFC-8 (~1.06 × 10^−4^ S·cm^−1^), a value indicating semiconductivity (~10^−9^-10^2^ S·cm^−1^) [46]. Considering this fact and possible numerical inaccuracy due to the rather small band gap value, the last two models were dropped in the subsequent investigations. Furthermore, as the other five models resulting in small band gaps give rise to nearly identical total energy and similar band structures, a-mod1 (Figure 5(e)) was chosen as a representative for the analyses of the electronic structure. As shown in Figure 5(e), the Zn-S^2-^ molecule in a-mod1 lies at the center of the channel parallel to the *a*-axis of the PFC-8 crystal. As shown in Figure 5(h) and Figure S17, the bands consisting mainly of the S-2p and C2-2p orbitals of the Zn-S^2-^ molecule are located in energy at the upper part of the band gap of PFC-8, leading to a Fermi level very close to the bottom of the conduction bands and a very small band gap *E*_*g*_ of Zn-S@PFC-8. The band state at Γ_3_′ (Figure 5(f)) consisting of a molecular orbital of the intercalated Zn-S^2-^ molecule describes the S-C p-p antibonding interaction and a small C-C p-p bonding interaction. The band state at Γ_2_′ (Figure S18) has a similar property. The energy band denoted by the Γ_1_′ state (Figure 5(h)) originates from the Cl1-Ni-Cl1 p-d-p antibonding molecular orbital from the PFC-8 framework and lies ~0.54 eV below the Fermi level. The CBM state (Γ_4_′) of a-mod1 is mainly composed of the orbitals from the PFC-8 framework (Figure 5(g) and Figure S18).

## 3. Discussion

Our calculation, for the first time, gives clear evidence that the bands (Γ_2_′ and Γ_3_′) due to the intercalated molecular orbitals lie in the gap of the host MOF framework, and in particular, they lie very close to the conduction band edge in the present case. The latter feature is similar to that of n-type semiconductors. The approaching of the dopant bands from the Zn-S^2-^ molecule to the conduction band makes it much easy to excite electrons from the dopant bands to the conduction bands by photons in a wide range of frequencies or even phonons, which increases largely the charge carrier concentration in the conduction band, and thus leads to a dramatic increase of the conductivity of the MOF material. Obviously, the Zn-S^2-^ molecule plays the role of electron donors as in the semiconductors. However, because of the large size, the complex structure, and the dynamics of the intercalants, Zn-S^2-^, the charge transfer mechanism may be more complicated than that in simple semiconductors such as phosphorus-doped silicon. As one can see from Figure 5(h), the conduction bands have in general very small bandwidths, which implies large effective masses of the charge carriers and thus renders the Zn-S@PFC-8 a semiconducting behavior instead of a metallic one. Considering the structural similarity among the intercalated MOF materials, we expect that the mechanism proposed for Zn-S@PFC-8 can also be applied to other semiconducting or conducting MOF materials. Our work clearly revealed two crucial factors for improving the electrical conductivity of a MOF material: (a) to increase the charge carrier concentration by intercalating suitable guest molecules and (b) to increase the dispersion of the conduction bands of the MOF framework by optimizing the interaction between the ligands and the metal ions.

In summary, the single-crystal conductivity of cationic MOF PFC-8 can be increased by five orders of magnitude through introducing anion electron-donor molecules inside pores. The electrostatic interaction plays an important role in stabilizing the intercalated small molecules. The guest molecules establish a new carrier transport route in an original insulated MOF and therefore drastically increase the electrical conductivity of the host framework. Compared with the usually adopted method of infiltrating neutral guests in neutral MOFs for conductivity improvement, the strategy presented here only needs a small number of guest molecules, capable of retaining the intrinsic porosity of frameworks. Meanwhile, the electrostatic interaction restrains guest molecules from leaching out of the porous framework, guaranteeing a reliable performance on conductivity. This work not only provides insight into the conductive mechanism in a host-guest system but also opens up a new pathway for regulating the conductivity of MOFs for broad practical applications.

## 4. Materials and Methods

### 4.1. Materials

Unless otherwise mentioned, all reagents and solvents were purchased from commercial sources and used as received without further purification: 1,4-Bis(4H-1,2,4-triazol-4-yl) benzene (97%, Jinan Henghua Technology Co., Ltd), 1,4-Di(1H-pyrazol-4-yl) benzene (95%, Adamas Reagent Co., Ltd), NiCl_2_·6H_2_O (99.0%, Adamas Reagent Co., Ltd), N,N-dimethylformamide (99.0% Sinopharm chemical Reagent Co., Ltd), triethylamine (99.5% Sinopharm chemical Reagent Co., Ltd), hydrochloric acid (36.0%-38.0% Sinopharm chemical Reagent Co., Ltd), and acetone (99.9% Sinopharm chemical Reagent Co., Ltd).

### 4.2. Fabrication of PFC-8 for Single-Crystal X-Ray Diffraction Analysis

The single crystal of PFC-8 was synthesized according to the previous report [30] with minor modifications. In brief, NiCl_2_·6H_2_O (16 mg) and 1,4-Bis(4H-1,2,4-triazol-4-yl) benzene (BTAB) (10 mg) in 2 mL of N,N-dimethylformamide (DMF) solution and 0.6 mL of water charged in a vial were ultrasonicated for a few minutes and followed with the addition of 0.43 mL triethylamine. The vial was heated in a 130°C oven for 16 h. The obtained crystals were further washed with 12 M HCl and DMF mixture solution (*v* : *v* = 2 : 1) twice and then with acetone three times to obtain a single crystal suitable for X-ray diffraction.

### 4.3. Fabrication of PFC-9 (Powdery Sample)

1,4-Di(1H-pyrazol-4-yl) benzene (H_2_DPB) in 15 mL DMF was ultrasonicated for 10 minutes and preheated at 90°C in a glass vial for 3 hours until completely dissolved. Ni(CH_3_COO)_2_·4H_2_O (125 mg) was added and ultrasonicated for 10 minutes. The obtained solution was transferred to five 10 mL glass vials (3 mL for each) and heated in a 120°C oven for 24 h. The obtained brown powdery crystals were further washed with fresh DMF and acetone three times, respectively.

### 4.4. Fabrication of Zn-S@PFC-8

The powdery crystals of PFC-8 (60 mg) and Zn-S·TA (189 mg) were washed in 63 mL of acetone for 18 h at room temperature. The obtained brown powdery crystals were further washed with fresh acetone many times until the supernatant is colorless.

### 4.5. Fabrication of Zn-S@PFC-9

The powdery crystals of PFC-9 (60 mg) and Zn-S·TA (189 mg) were washed in 63 mL of acetone for 18 h at room temperature. The obtained brown powdery crystals were further washed with fresh acetone many times until the supernatant is colorless.

### 4.6. Fabrication of the Pellets of PFC-9 and Zn-S@PFC-9

10 mg of each powdery sample (PFC-9 and Zn-S@PFC-9) was added into tablet press mould (4 mm × 4 mm, Base Material: Cr12MoV, hardness: HRC60-HRC62) and forced with 0.5 MPa pressure for 30 s. The size of the obtained pellet is about 4 mm × 4 mm × 0.5 mm.

### 4.7. Instrumentation and Characterizations

Powder X-ray diffraction (PXRD) patterns of the samples were collected on Rikagu Miniflex 600 Benchtop X-ray diffraction instrument. The N_2_ sorption isotherms were measured using ASAP 2460 from Micromeritics Co. Ltd. Scanning electron microscopy (SEM) images were obtained using a HITACHI SU8000 field emission scanning electron microscope. UV-Vis diffuse reflectance spectroscopy (DRS) was performed with a Cary 500 UV-Vis spectrophotometer. After using BaSO_4_ as the blank reference, powder samples were directly loaded in the cell for tests without further dilution by BaSO_4_. X-ray photoelectron spectroscopy (XPS) measurements were carried out using an ESCALAB 250Xi spectrometer (Thermo Fisher Co. Ltd) with monochromatic Al K*α* radiation (*E* = 1486.2 eV) in combination with sputter etching by an Ar ion beam to remove the surface oxide and containments. Tantalum pentoxide was used as a reference for XPS etching depth. C 1s peak at 284.8 eV was used as the charge correction reference. Ion chromatography measurements were carried out using Metrohm 883 at room temperature. XAFS spectra were measured at the BL14W1 beamline station of the Shanghai Synchrotron Radiation Facility, China. EDS (Energy Dispersive Spectrometer) data were collected by a field emission scanning electron microscope, FESEM (JSM6700-F). Temperature-dependent magnetization data were collected by the Physical Property Measurement System (PPMS) (model 6000).

### 4.8. Single-Crystal Conductivity Measurements

Two-contact probe devices were fabricated by attaching two aluminum electrodes on the two ends of PFC-8 (or Zn-S@PFC-8) single crystals along the long axis on silicon substrates. The voltage and current data were then collected by a computer connected with a semiconductor parametric analyzer (Hewlett Packard 4155B).

### 4.9. Powdery Sample Conductivity Measurements

The powdery samples of PFC-9 and Zn-S@PFC-9 were pressed to pellets for measurement. Two-probe devices were attached with two copper electrodes onto both the up and bottom surfaces of the pellet. The voltage and current data were collected by a computer connected with a semiconductor parameter analyzer (Hewlett Packard 4155B).

## Data Availability

More detailed experimental methods are available in Supplementary Materials, including computational details, CV, and EDS elemental mapping.

## Supplementary Materials

Supplementary Materials Figure S1: (a) the PXRD patterns of PFC-8 and PFC-8 simulated. (b) UV-Vis-NIR spectra of PFC-8 after being immersed in DMF solution with different concentrations of Zn-S·TA. Figure S2: (a) NaCl solution standard curve. (b) The ingredient changes of supernatant during the ion-exchange process in 9 hours monitored by ion chromatography. Figure S3: UV-Vis-NIR spectra of Zn-S·TA. Figure S4: (a) X-ray photoelectron spectroscopy (XPS) spectra of PFC-8 (black) and Zn-S@PFC-8 (red). (b) XPS spectra of Zn-S@PFC-8 after etching at different depths (0 nm, 5 nm, 10 nm, 25 nm, and 50 nm). Table S1: Atomic Absorption (AA) spectroscopy of the single crystal of PFC-8 and Zn-S@PFC-8 (m1 = molecular weight of Zn-S^2-^; m2 = molecular weight of PFC-8). Table S2: Ni K-edge EXAFS curve fitting results of PFC-8 and Zn-S@PFC-8. Table S3: EDS results of the single crystal of Zn-S@PFC-8. Figure S5: the linear fitting of voltage and current of PFC-8 (single crystal) and Zn-S@PFC-8. Table S4: the size and conductivity of the single-crystal samples. Figure S6: (a, b) three parallel tests of *I*–*V* of single-crystal PFC-8 and single-crystal Zn-S@PFC-8. Figure S7: EXAFS spectra of PFC-8 and Zn-S@PFC-8. Inset: the q space curves of PFC-8 and Zn-S@PFC-8. Figure S8: EXAFS fitting curves of Zn-S@PFC-8 and PFC-8. The blue lines show experimental data, and the yellow lines show the best fitting result, and sky blue lines show the fitting range. Figure S9: the schematic presentation of the structures of PFC-8 and PFC-9. Table S5: the AA analyses of the square shape pellet samples. Table S6: the size and conductivity of the square shape pellet samples (length = wide). Figure S10: (a) the PXRD patterns of PFC-9, Zn-S@PFC-9, and PFC-9 simulated. (b) The linear fitting of voltage and current of PFC-9 and Zn-S@PFC-9. Figure S11: XPS spectra of Zn-S@PFC-9 with different etching depths (0 nm, 5 nm, 10 nm, 25 nm, and 50 nm). Figure S12: (a, b) the cyclic voltammetry of PFC-8 and Zn-S@PFC-8 and (c, d) PFC-9 and Zn-S@PFC-9. Table S7: experimentally reported crystal structure of PFC-8. The atom sites with half occupancy are highlighted in red. Table S8: calculated static total energy of 16 models of PFC-8 considering the orientation of the benzene ring. The mod-6 and mod-11 with the highest space group are highlighted. Note that these two models are essentially the same one and mod-11 is used in the following calculation. Table S9: calculated total energy and bandgaps for seven models of Zn-S@PFC-8. Figure S13: temperature-dependent magnetization of PFC-8 and Zn-S@PFC-8. Figure S14: (a) experimental reported crystal structure of PFC-8. The red dash circles represent the benzene ring with half-occupied C and H atoms. (b) Two possible orientations, R and L, for each benzene ring. (c) Structure model of *Pnma* PFC-8. Figure S15: the screening of all possible structures due to the half-occupied Cl2 atoms. (a) Calculated total energy of 70 structure models. (b) The model with the lowest energy, *P*2_1_2_1_2_1_ PFC-8. Note that, for the convenience of building the models with doped Zn-S^2-^ molecules, the unit cell origin of *P*2_1_2_1_2_1_ PFC-8 is shifted by the vector *p* = 0.5*a*, as presented in Figure 5(a) in the text. Figure S16: structure models for Zn-S@PFC-8. The initial models (a-g) and the corresponding optimized models (h-n) for a-mod1, c-mod1, b-mod1, b-mod2, b-mod3, b-mod4, and b-mod5, respectively. The prefix of these models are named after the orientation of the intercalated Zn-S^2-^ molecule. Figure S17: calculated DOS for (a) *P*2_1_2_1_2_1_ PFC-8 and (b) a-mod1 of Zn-S@PFC-8 at the energy range of -3.5 to 2 eV. The Fermi energy level is set at 0 eV. Figure S18: selected orbitals for a-mod1 Zn-S@PFC-8. Table S10: summary of the data for some conductive MOFs.
