# Supplementary material for: Harnessing Electrostatic Interactions for Enhanced Conductivity in Metal-Organic Frameworks
Source: Research (Wash D C). 2021 Oct 21;2021:9874273. doi: 10.34133/2021/9874273 (PMC8556649; doi:10.34133/2021/9874273)
Supplement: Supplementary Materials — Figure S1: (a) the PXRD patterns of PFC-8 and PFC-8 simulated. (b) UV-Vis-NIR spectra of PFC-8 after being immersed in DMF solution with different concentrations of Zn-S·TA. Figure S2: (a) NaCl solution standard curve. (b) The ingredient changes of supernatant during the ion-exchange process in 9 hours monitored by ion chromatography. Figure S3: UV-Vis-NIR spectra of Zn-S·TA. Figure S4: (a) X-ray photoelectron spectroscopy (XPS) spectra of PFC-8 (black) and Zn-S@PFC-8 (red). (b) XPS spectra of Zn-S@PFC-8 after etching at different depths (0 nm, 5 nm, 10 nm, 25 nm, and 50 nm). Table S1: Atomic Absorption (AA) spectroscopy of the single crystal of PFC-8 and Zn-S@PFC-8 (m1 = molecular weight of Zn-S2-; m2 = molecular weight of PFC-8). Table S2: Ni K-edge EXAFS curve fitting results of PFC-8 and Zn-S@PFC-8. Table S3: EDS results of the single crystal of Zn-S@PFC-8. Figure S5: the linear fitting of voltage and current of PFC-8 (single crystal) and Zn-S@PFC-8. Table S4: the size and conductivity of the single-crystal samples. Figure S6: (a, b) three parallel tests of I–V of single-crystal PFC-8 and single-crystal Zn-S@PFC-8. Figure S7: EXAFS spectra of PFC-8 and Zn-S@PFC-8. Inset: the q space curves of PFC-8 and Zn-S@PFC-8. Figure S8: EXAFS fitting curves of Zn-S@PFC-8 and PFC-8. The blue lines show experimental data, and the yellow lines show the best fitting result, and sky blue lines show the fitting range. Figure S9: the schematic presentation of the structures of PFC-8 and PFC-9. Table S5: the AA analyses of the square shape pellet samples. Table S6: the size and conductivity of the square shape pellet samples (length = wide). Figure S10: (a) the PXRD patterns of PFC-9, Zn-S@PFC-9, and PFC-9 simulated. (b) The linear fitting of voltage and current of PFC-9 and Zn-S@PFC-9. Figure S11: XPS spectra of Zn-S@PFC-9 with different etching depths (0 nm, 5 nm, 10 nm, 25 nm, and 50 nm). Figure S12: (a, b) the cyclic voltammetry of PFC-8 and Zn-S@PFC-8 and (c, d) PFC-9 and Zn-S [file 9874273.f1.doc]

Supplementary Materials for

**Harnessing Electrostatic Interactions for Enhanced Conductivity in Metal-Organic Frameworks**

An-An Zhang, Xiyue Cheng, Xu He, Wei Liu, Shuiquan Deng, Rong Cao* and Tian-Fu Liu*

Dr. A. A. Zhang, Prof. R. Cao

Department of Chemistry, School of Chemistry and Materials Science, University of Science and Technology of China, Hefei, Anhui 230026, P. R. China.

Dr. A. A. Zhang, Dr. X. Y Cheng, Dr. X. He, Prof. W. Liu, Prof. S. Q. Deng, Prof. T. F. Liu, Prof. R. Cao

State Key Laboratory of Structural Chemistry, Fujian Institute of Research on the Structure of Matter, Chinese Academy of Sciences, Fuzhou, Fujian 350002, P. R. China.
E-mail: Tian-Fu Liu [tfliu@fjirsm.ac.cn](mailto:tfliu@fjirsm.ac.cn) Rong Cao: [rcao@fjirsm.ac.cn](mailto:rcao@fjirsm.ac.cn)

Prof. S. Q. Deng, Prof. T. F. Liu, Prof. R. Cao

University of the Chinese Academy of Sciences, Beijing 100049, P. R. China.

Contents

[**Computational Details** 5](#__RefHeading___Toc72765870)

[**Figure S1.** a) The PXRD patterns of PFC-8 and PFC-8 simulated. b) UV-Vis-NIR spectra of PFC-8 after being immersed in DMF solution with different concentration of Zn-S·TA. 7](#__RefHeading___Toc72765871)

[**Figure S2.** (a) NaCl solution standard curve. (b) The ingredient changes of supernatant during the ion-exchange process in 9 hours monitored by ion chromatography. 8](#__RefHeading___Toc72765873)

[**Figure S3.** UV-Vis-NIR spectra of Zn-S·TA. 8](#__RefHeading___Toc72765874)

[**Figure S4.** (a). X-ray photoelectron spectroscopy (XPS) spectra of PFC-8 (black) and **Zn-S@PFC-8** (red). (b). XPS spectra of **Zn-S@PFC-8** after etching at different depths (0 nm, 5 nm, 10 nm, 25 nm, 50 nm). 9](#__RefHeading___Toc72765875)

[**Table S1.** Atomic Absorption (AA) Spectroscopy of the single crystal of PFC-8 and **Zn-S@PFC-8** (m_1_= molecular weight of Zn-S^2-^, m_2_= molecular weight of PFC-8). 10](#__RefHeading___Toc72765876)

[**Table S2.** Ni K-edge EXAFS curve fitting results of PFC-8 and **Zn-S@PFC-8**. 10](#__RefHeading___Toc72765877)

[**Table S3.** EDS results of the single crystal of **Zn-S@PFC-8** 11](#__RefHeading___Toc72765878)

[**Figure S5.** The linear fitting of voltage and current of PFC-8 (single crystal) and **Zn-S@PFC-8** 12](#__RefHeading___Toc72765879)

[**Table S4.** The size and conductivity of the **single-crystal** samples. 12](#__RefHeading___Toc72765880)

[**Figure S6.** a) b) Three parallel tests of I–V of single crystal PFC-8 and single crystal **Zn-S@PFC-8**. 13](#__RefHeading___Toc72765881)

[**Figure S7.** EXAFS spectra of PFC-8 and **Zn-S@PFC-8**. Inset: The q spaces curves of PFC-8 and **Zn-S@PFC-8** 14](#__RefHeading___Toc72765882)

[**Figure S8.** EXAFS fitting curves of **Zn-S@PFC-8** and PFC-8. The blue lines show experimental data, and the yellow lines show the best fitting result, and sky blue lines show the fitting range. 14](#__RefHeading___Toc72765883)

[**Figure S9.** The schematic presentation of the structures of PFC-8 and PFC-9. 15](#__RefHeading___Toc72765884)

[**Table S5.** The AA analyses of **the square shape pellet** samples. 15](#__RefHeading___Toc72765885)

[**Table S6.** The size and conductivity of **the square shape pellet samples** (Length = wide) 15](#__RefHeading___Toc72765886)

[**Figure S10** a) The PXRD patterns of PFC-9, Zn-S@PFC-9 and PFC-9 simulated. b) The linear fitting of voltage and current of PFC-9, and Zn-S@PFC-9. 16](#__RefHeading___Toc72765887)

[**Figure S11.** XPS spectra of Zn-S@PFC-9 with different etching depths (0 nm, 5 nm, 10 nm, 25 nm, 50 nm). 17](#__RefHeading___Toc72765888)

[**Figure S12. a), b)** The cyclic voltammetry of PFC-8, **Zn-S@PFC-8**, **c), d)** PFC-9, and **Zn-S@PFC-9**. 18](#__RefHeading___Toc72765889)

[**Table S7.** Experimentally reported crystal structure of PFC-8. The atom sites with half occupancy are highlighted in red. 19](#__RefHeading___Toc72765890)

[**Table S8**. Calculated static total energy of 16 models of PFC-8 considering the orientation of the benzene ring. The mod-6 and mod-11 with the highest space group are highlighted. Note that these two models are essentially the same one and mod-11 is used in the following calculation. 20](#__RefHeading___Toc72765891)

[**Table S9.** Calculated total energy and bandgaps for seven models of **Zn-S@PFC-8**. 21](#__RefHeading___Toc72765892)

[**Figure S13.** Temperature-dependent magnetization of PFC-8 and **Zn-S@PFC-8**. 22](#__RefHeading___Toc72765893)

[**Figure S14.** (a) Experimental reported crystal structure of PFC-8. The red dash circles represent the benzene ring with half occupied C and H atoms. (b) Two possible orientation, R and L, for each benzene ring. (c) Structure model of *Pnma* PFC-8. 23](#__RefHeading___Toc72765894)

[**Figure S15** The screening of all possible structures due to the half occupied Cl2 atoms. (a) Calculated total energy of 70 structure models. (b) The model with the lowest energy, *P*2_1_2_1_2_1_ PFC-8. Note that, for the convenience of building the models with doped Zn-S^2-^ molecules, the unit cell origin of *P*2_1_2_1_2_1_ PFC-8 are shifted by the vector ***p***=0.5***a***, as presented in **Figure 5a** in the text. 24](#__RefHeading___Toc72765895)

[**Figure S16**. Structure models for **Zn-S@PFC-8**. The initial models (a-g) and the corresponding optimized models (h-n) for *a*-mod1, *c*-mod1, *b*-mod1, *b*-mod2, *b*-mod3, *b*-mod4 and *b*-mod5, respectively. The prefix of these models are named after the orientation of the intercalated Zn-S^2-^ molecule. 25](#__RefHeading___Toc72765896)

[**Figure S17.** Calculated DOS for (a) *P*2_1_2_1_2_1_ PFC-8 and (b) *a*-mod1 of **Zn-S@PFC-8** at the energy range of -3.5 to 2 eV, The Fermi energy level is set at 0 eV. 26](#__RefHeading___Toc72765897)

[**Figure S18.** Selected orbitals for a-mod1 Zn-S@PFC-8 27](#__RefHeading___Toc72765898)

[**Table S10.** Summary of the data for some conductive MOFs. 28](#__RefHeading___Toc72765899)

[**Reference** 29](#__RefHeading___Toc72765900)

# Computational Details

The structural and electronic properties of PFC-8 and Zn-S@PFC-8 were calculated within the framework of density functional theory (DFT)^1,2^ by using the Vienna *ab-initio* simulation package (VASP)^3-5^ with the projector augmented wave (PAW) method.^6^ The generalized gradient approximation (GGA) within the Perdew-Burke-Ernzerhof (PBE) type exchange-correlation potentials^7^ was used throughout this work. The employed PAW-PBE pseudopotentials^8^ of elements Zn, Ni, Cl, S, N, C and H treat 3*d*^10^4*s*^2^, 3*p*^6^3*d*^8^4*s*^2^, 3*s*^2^3*p*^5^, 3*s*^2^3*p*^4^, 2*s*^2^2*p*^3^, 2*s*^2^2*p*^2^ and 1*s* as the valence states, respectively. To account for the strong electron correlation effects on the *d* shells of Ni atoms, the DFT + *U* scheme^9^ in Dudarev’s approach^10^ with an effective Coulomb parameter *U_eff_* = 2.5 eV is used. The plane wave cutoff energy for the expansion of wave functions was set at 550 eV with dense *k*-point mesh, 2 × 5 × 3 and 2 × 2 × 3, for the models of PFC-8 and Zn-S@PFC-8, respectively. The conjugate-gradient algorithm as implemented in the VASP code was used in all structural relaxation. In this work, both the cell volume and the atomic positions were all allowed to relax to minimize the internal forces. Excellent convergence of the energy differences (0.1 eV) and stress tensors (0.05 eV) was achieved. From the experimental crystal structure of PFC-8 (Table S7), several half occupied atomic sites can be found, i.e. C3, C4, H2, H3 atoms on the benzene ring in the MOF framework and Cl2 atoms in channels (Figure S14). To avoid the rough virtual crystal approximation, a structure model with no fractional occupancy of atomic sites for MOF PFC-8 is required. There are 4 benzene rings in one unit cell of PFC-8 and each benzene ring has two possible orientation due to the half occupancy of C and H atoms, thus resulting
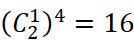
 possible structure models (Figure S14). However, the energy differences of the 16 models are so small (Table S8) that none of them can be picked out as the unique candidate model. For the convenience of calculation, the structure model with the highest space group, i.e. *Pnma*, was selected for the MOF framework. Based on this *Pnma* model, the half occupancy of the eight Cl2 atoms are further solved by generating and screening all
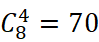
 possible structures (Figure S15). The total energy of these models are obtained by using the high-throughput approach as the computational engine. The structure model with space group *P*2_1_2_1_2_1_ (Figure 5a) is subsequently selected among the 70 models through energy optimizations and atomic coordinates as the initial structural model of PFC-8. As each Zn-S^2-^ molecule has two negative charges, it would replace two Cl^-^ ions during the incorporation for charge balance. Considering both the experimental molecular formula of Zn-S@PFC-8 (C_10_H_8_N_6_NiCl_1.986_(ZnS_10_C_6_)_0.007_) and the size effect of Zn-S^2-^ molecule, we have built 7 independent structure models (Figure S16) by doping one Zn-S^2-^ molecule into the PFC-8 supercell and replacing two Cl atoms simultaneously, thus resulting models containing 339 atoms with the formula of C_10_H_8_N_6_NiCl_1.83_(ZnS_10_C_6_)_0.083_.


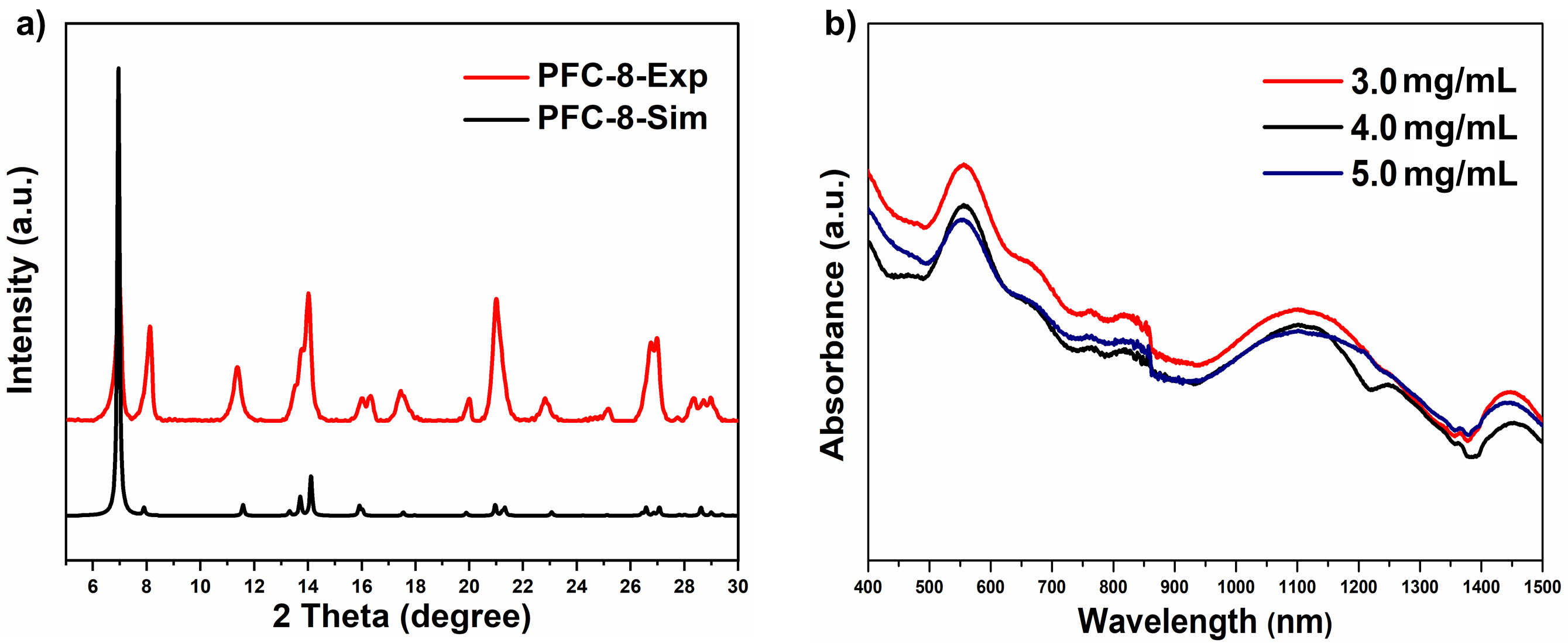


## **Figure S1.** a) The PXRD patterns of PFC-8 and PFC-8 simulated. b) UV-Vis-NIR spectra of PFC-8 after being immersed in DMF solution with different concentration of Zn-S·TA.

Zn-S·TA loading was conducted in DMF solution with different Zn-S·TA concentrations (3.0, 4.0 and 5.0 mg/mL) at 25 ℃. The obtained samples gave rise to almost identical UV-Vis-NIR spectra, illustrating that the concentration did not greatly change the loading amount. However, higher Zn-S·TA concentration dramatically detracted the porosity of PFC-8. To reconcile the conductivity and porosity, Zn-S^2-^ loading was conducted in 3.0 mg/mL Zn-S·TA solution in this study.


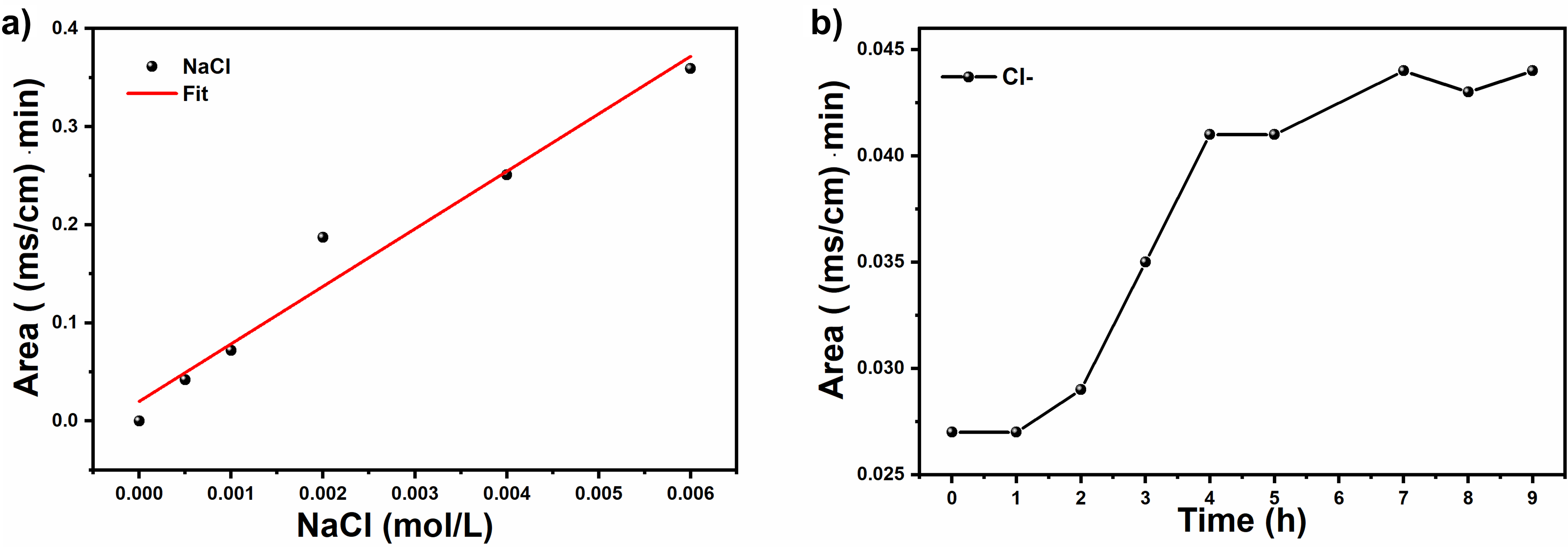


## **Figure S2.** (a) NaCl solution standard curve. (b) The ingredient changes of supernatant during the ion-exchange process in 9 hours monitored by ion chromatography.


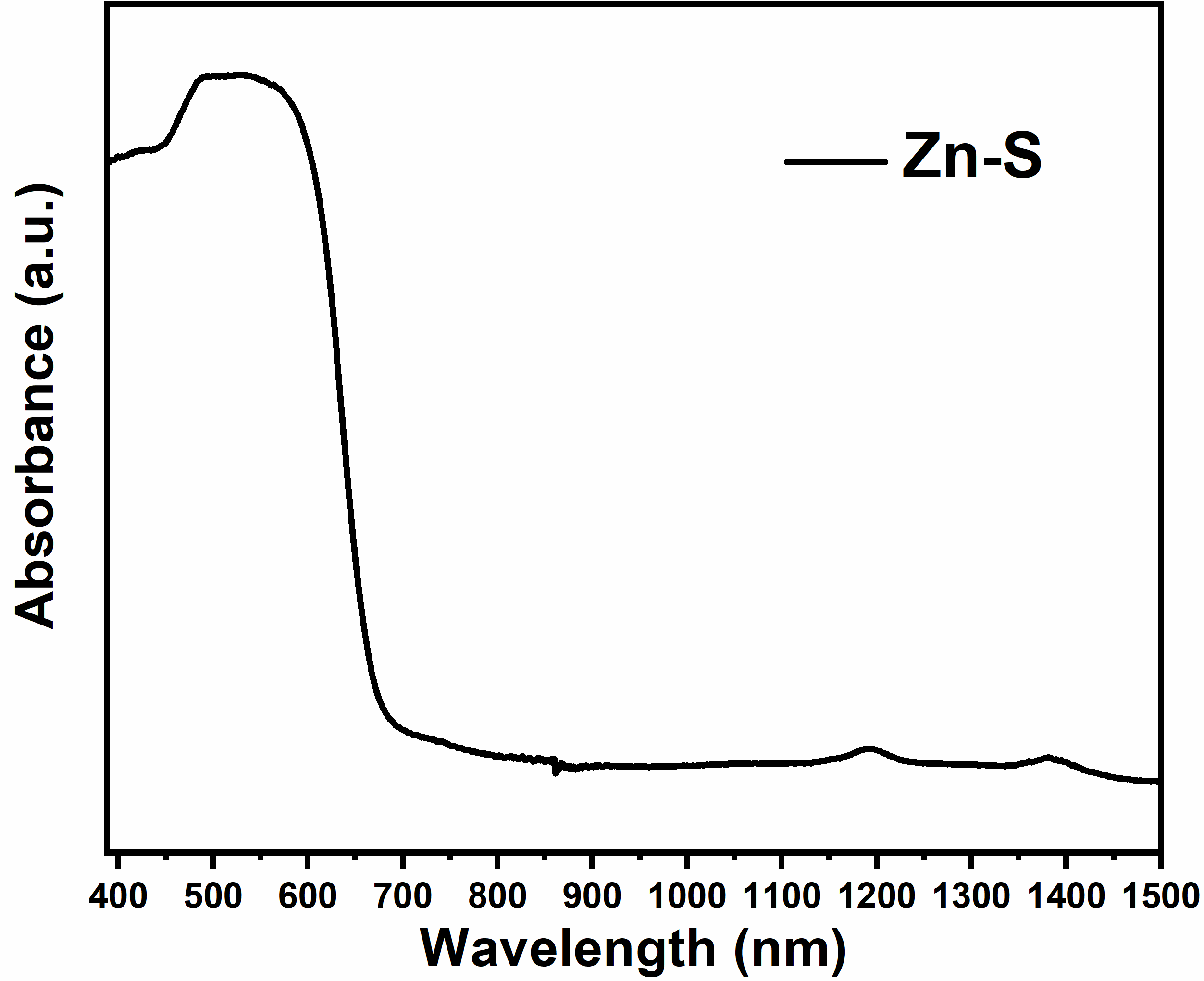


## **Figure S3.** UV-Vis-NIR spectra of Zn-S·TA.


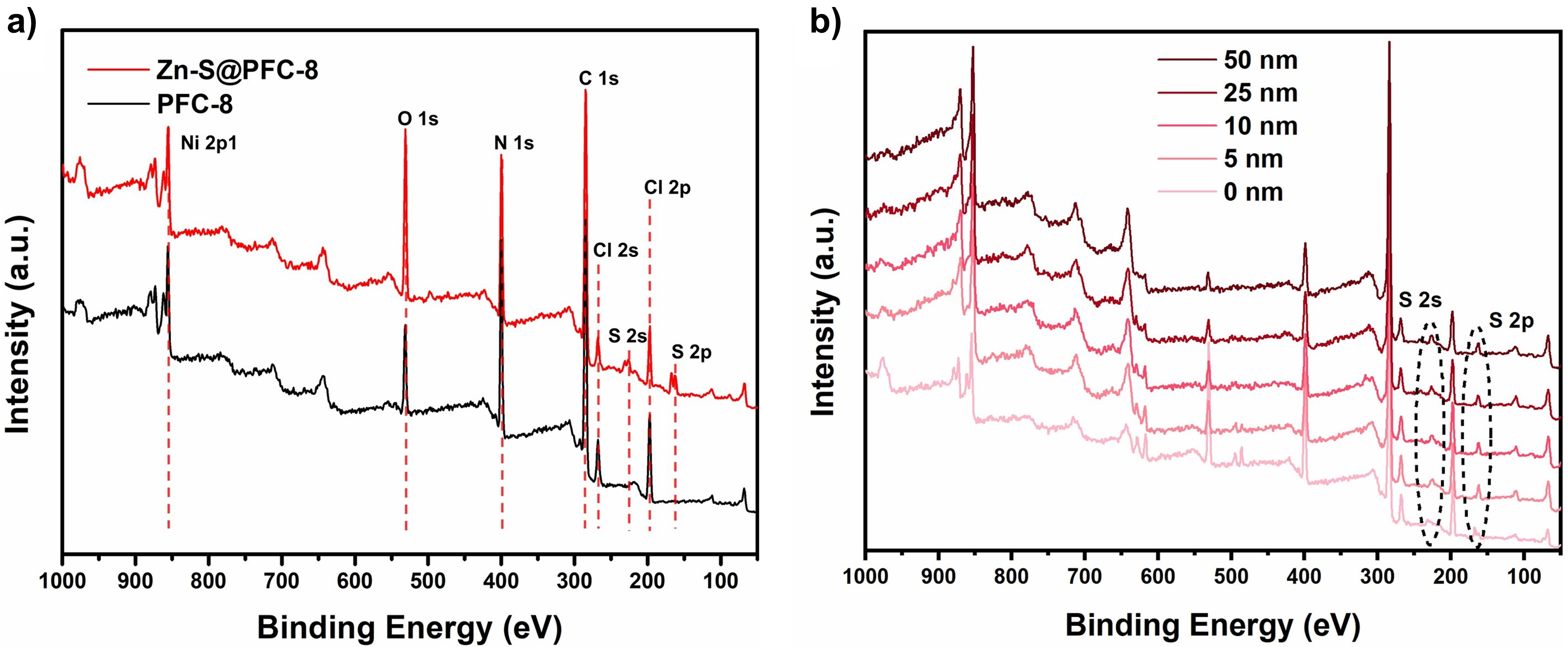


## **Figure S4.** (a). X-ray photoelectron spectroscopy (XPS) spectra of PFC-8 (black) and **Zn-S@PFC-8** (red). (b). XPS spectra of **Zn-S@PFC-8** after etching at different depths (0 nm, 5 nm, 10 nm, 25 nm, 50 nm).

## **Table S1.** Atomic Absorption (AA) Spectroscopy of the single crystal of PFC-8 and **Zn-S@PFC-8** (m_1_= molecular weight of Zn-S^2-^, m_2_= molecular weight of PFC-8).

| **Samples** | **Zn** | **Ni** | **Loading (m_1_/m_2_)** |
| --- | --- | --- | --- |
| **PFC-8** | 0% | 10.60% | 0% |
| **Zn-S@PFC-8** | 0.0767% | 10.05% | 0.9% |

**Calculation equation of loading amount:**


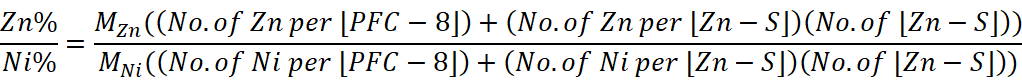


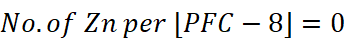


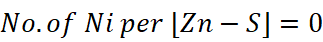


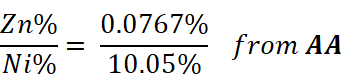


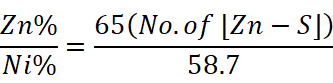


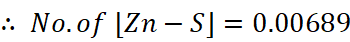


## **Table S2.** Ni K-edge EXAFS curve fitting results of PFC-8 and **Zn-S@PFC-8**.

| **Samples** | **Path** | **CN** | ***R* (Å)** | **σ^2^ (Å^2^)** | **R factor** |
| --- | --- | --- | --- | --- | --- |
| PFC-8 | Ni-N | 4 | 2.04 | 0.00621 | 0.02 |
|  | Ni-Cl | 2 | 2.41 | 0.00114 |  |
| **Zn-S@PFC-8** | Ni-N | 4 | 1.91 | 0.00621 | 0.02 |
|  | Ni-Cl | 2 | 2.18 | 0.00114 |  |

CN, coordination number; *R*, distance between absorber and backscatter atoms; *σ*^2^, Debye-Waller factor (a measurement of thermal and static disorder in absorber-scatter distances); R factor is used to value the goodness of the fitting.

## **Table S3.** EDS results of the single crystal of **Zn-S@PFC-8**


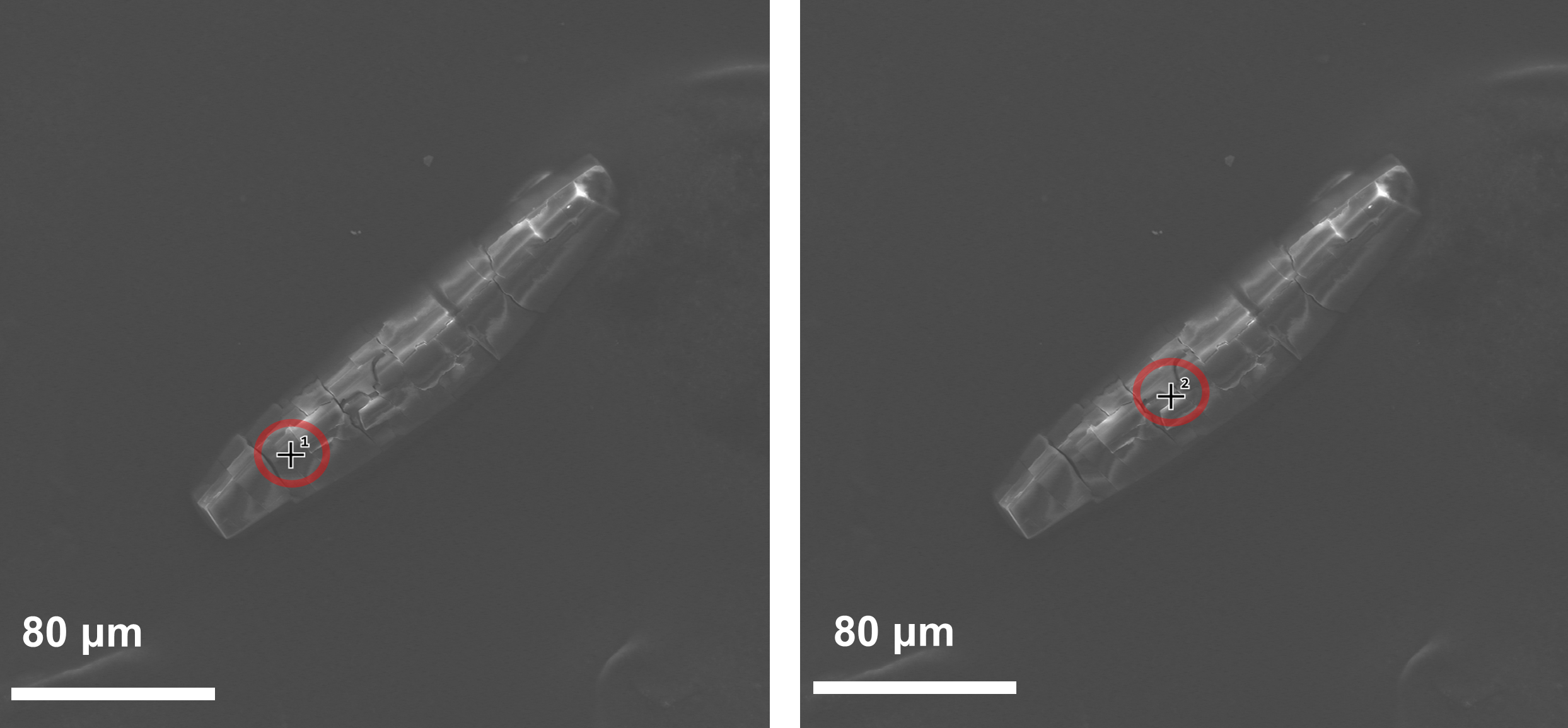


| **Element** | **Point** | **Ni** | **Cl** | **S** | **Zn** |
| --- | --- | --- | --- | --- | --- |
| **Weight (%）** | **1#** | 10.30 | 13.31 | **0.33** | **0.07** |
| **Atom (%)** |  | 2.75 | 5.88 | **0.16** | **0.02** |
| **Weight (%）** | **2#** | 30.83 | 38.83 | **0.73** | **0.20** |
| **Atom (%)** |  | 13.11 | 27.34 | **0.57** | **0.08** |


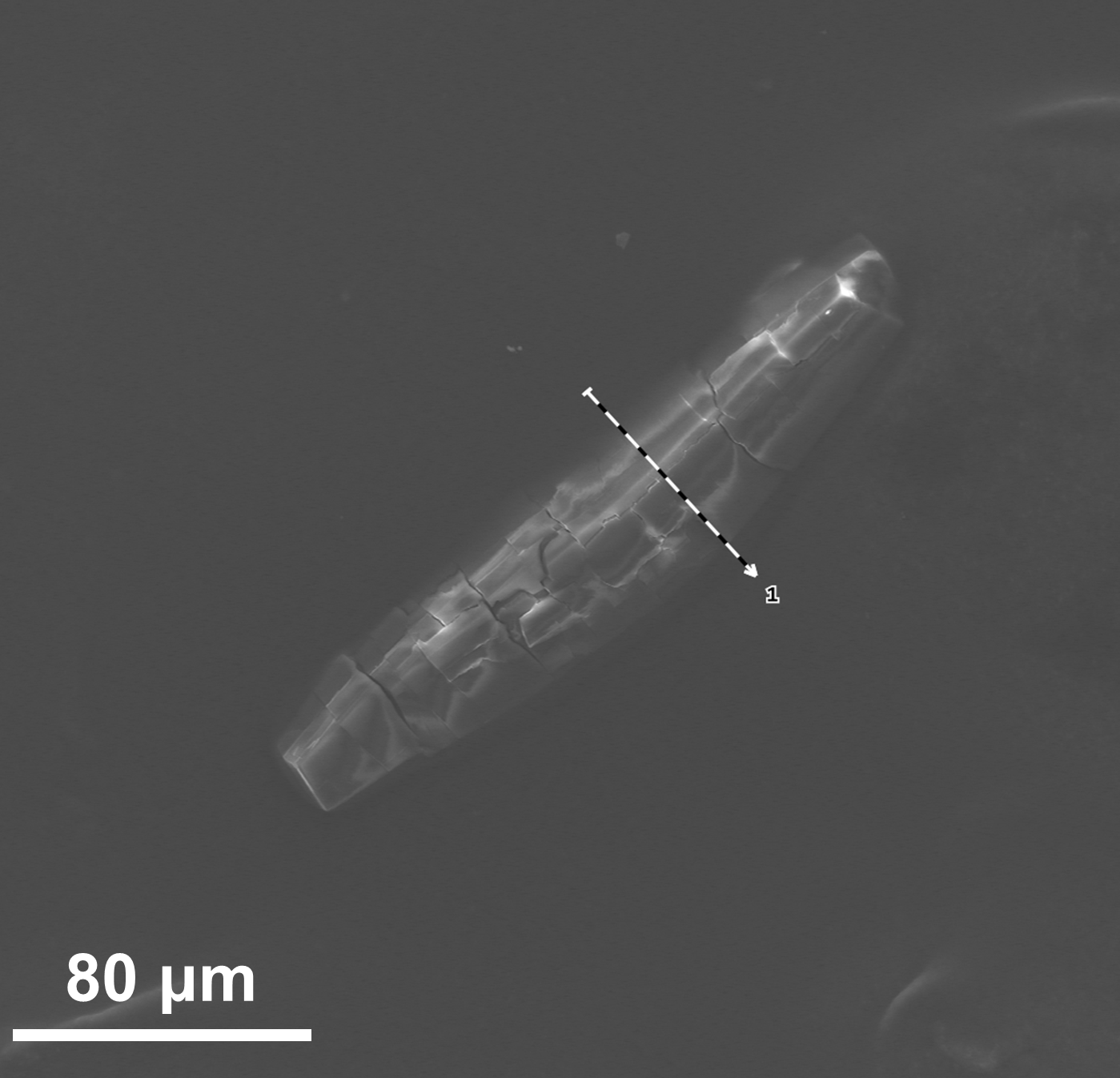


| **Element** | **Ni** | **Cl** | **S** | **Zn** |
| --- | --- | --- | --- | --- |
| **Weight (%）** | 5.14 | 5.22 | **0.17** | **0.03** |
| **Atom (%)** | 1.20 | 2.01 | **0.07** | **0.01** |

The EDS measurements conducted in point-, line-scan model gave rise to similar result about a loading amount of 0.7 mol%, which coincides with the data obtained from AA analysis shown in **Table S1**. Moreover, the Zn and S ratio (1:8) is close to the calculated result (1:10) based on the chemical formula, suggesting that Zn-S^2-^ molecules remain the structural integration upon loading process.


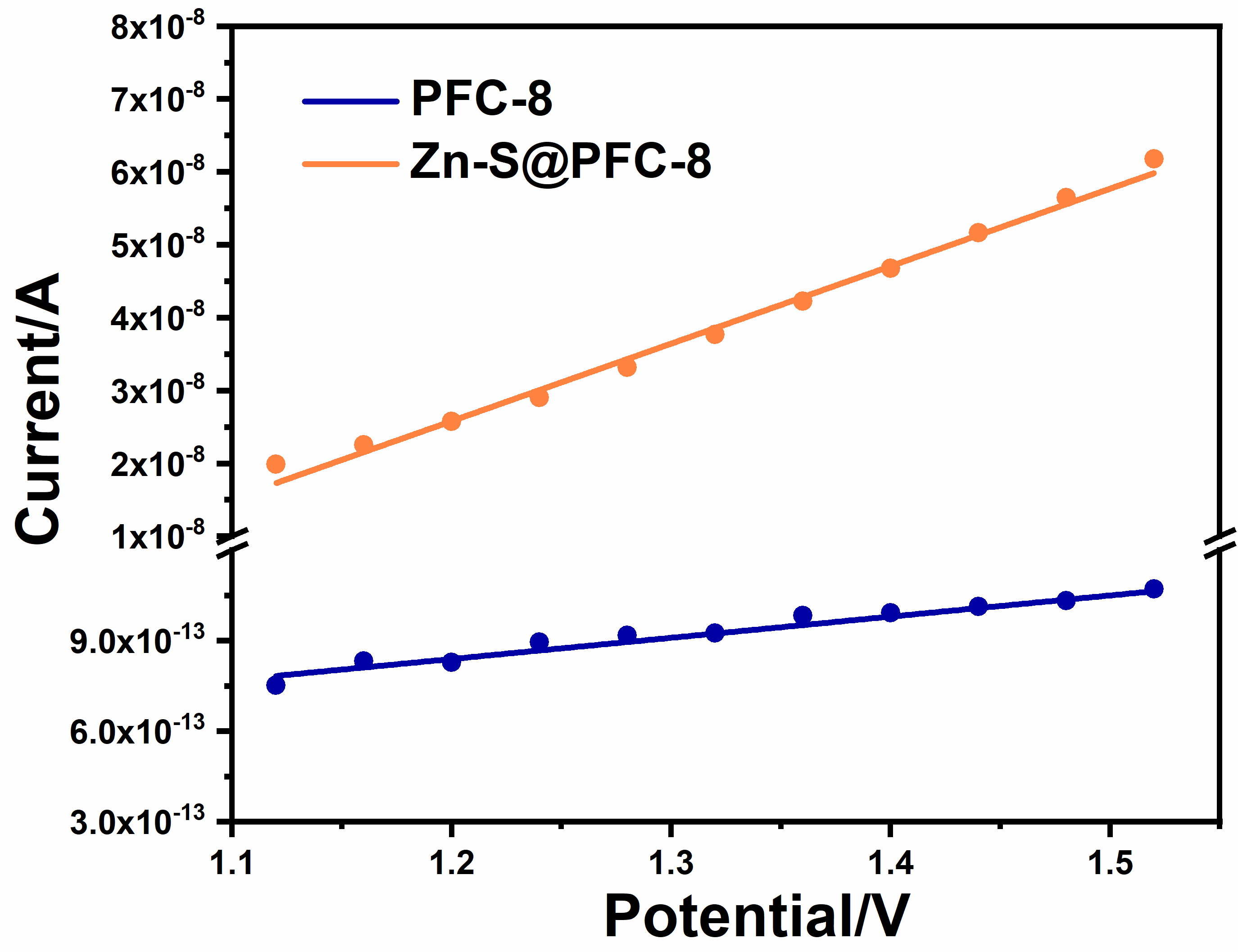


## **Figure S5.** The linear fitting of voltage and current of PFC-8 (single crystal) and **Zn-S@PFC-8**.

Note: Calculation equation of conductivity σ:


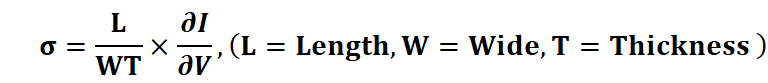


The nonlinear I-V curve at low voltage range is mainly caused by the large contact resistance. Therefore, only the data showing good linear relation was fitted to deduce a conductivity of material. Since both the positive and negative biases gave rise to similar I-V curves, only positive bias is selected for fitting.

## **Table S4.** The size and conductivity of the **single-crystal** samples.

| **Samples** | **Thickness (μm) (cm·10^-4^)** | **Length (μm) (cm·10^-4^)** | **Wide (μm) (cm·10^-4^)** | **Conductivity (S cm^-1^)** |
| --- | --- | --- | --- | --- |
| **PFC-8** | 18 | 173 | 67 | 1.01×10^-9^ |
| **Zn-S@PFC-8** | 18 | 120 | 66 | 1.06×10^-4^ |


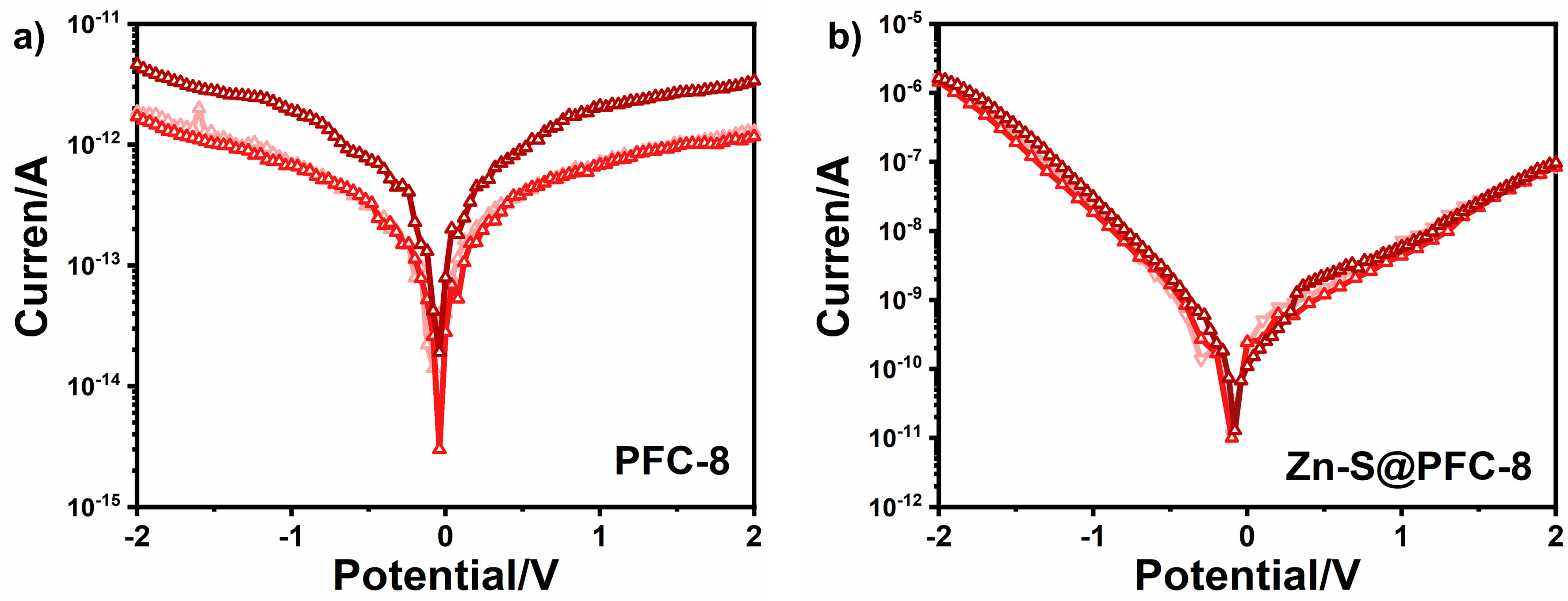


## **Figure S6.** a) b) Three parallel tests of I–V of single crystal PFC-8 and single crystal **Zn-S@PFC-8**.


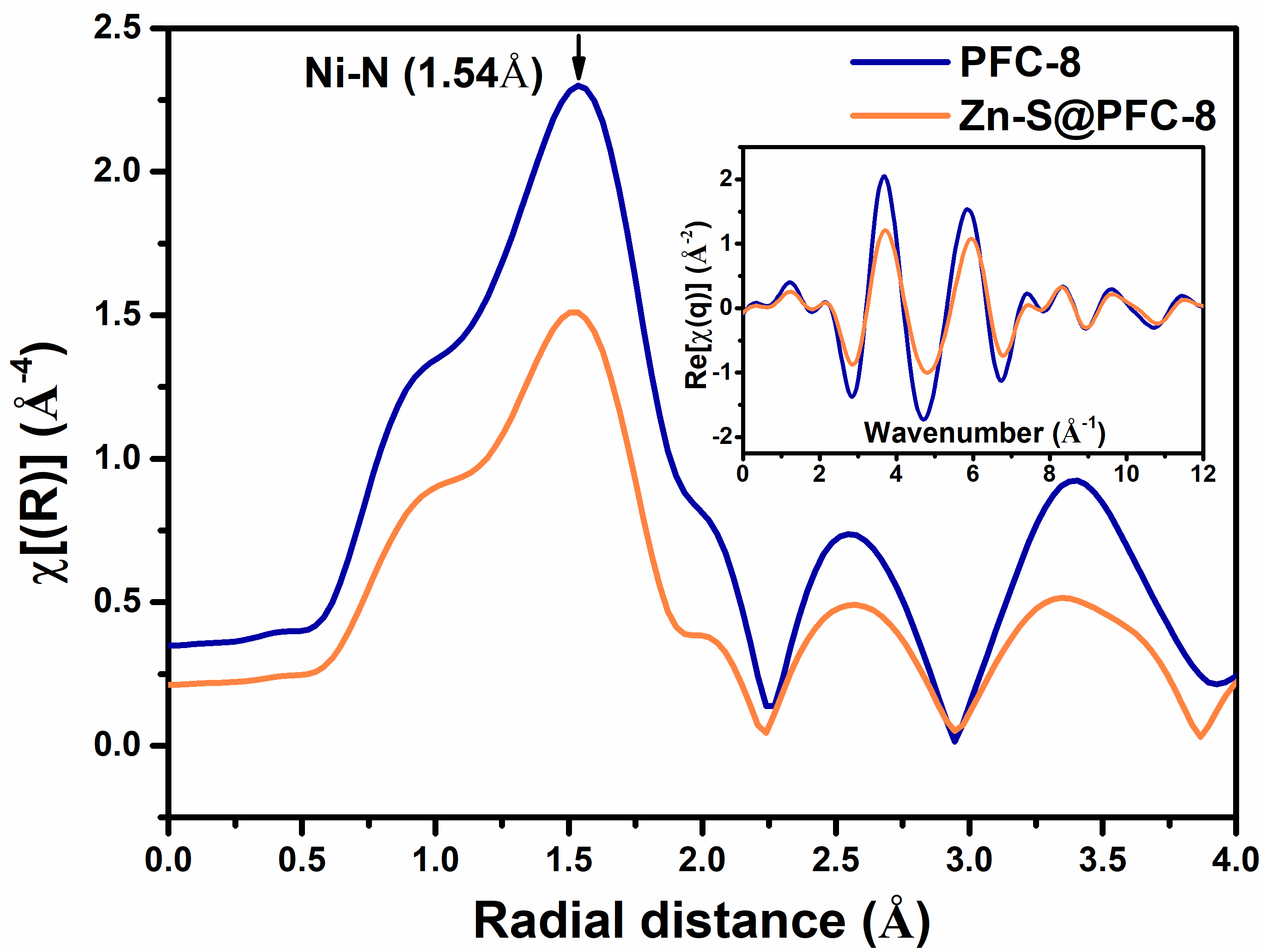


## **Figure S7.** EXAFS spectra of PFC-8 and **Zn-S@PFC-8**. Inset: The q spaces curves of PFC-8 and **Zn-S@PFC-8**


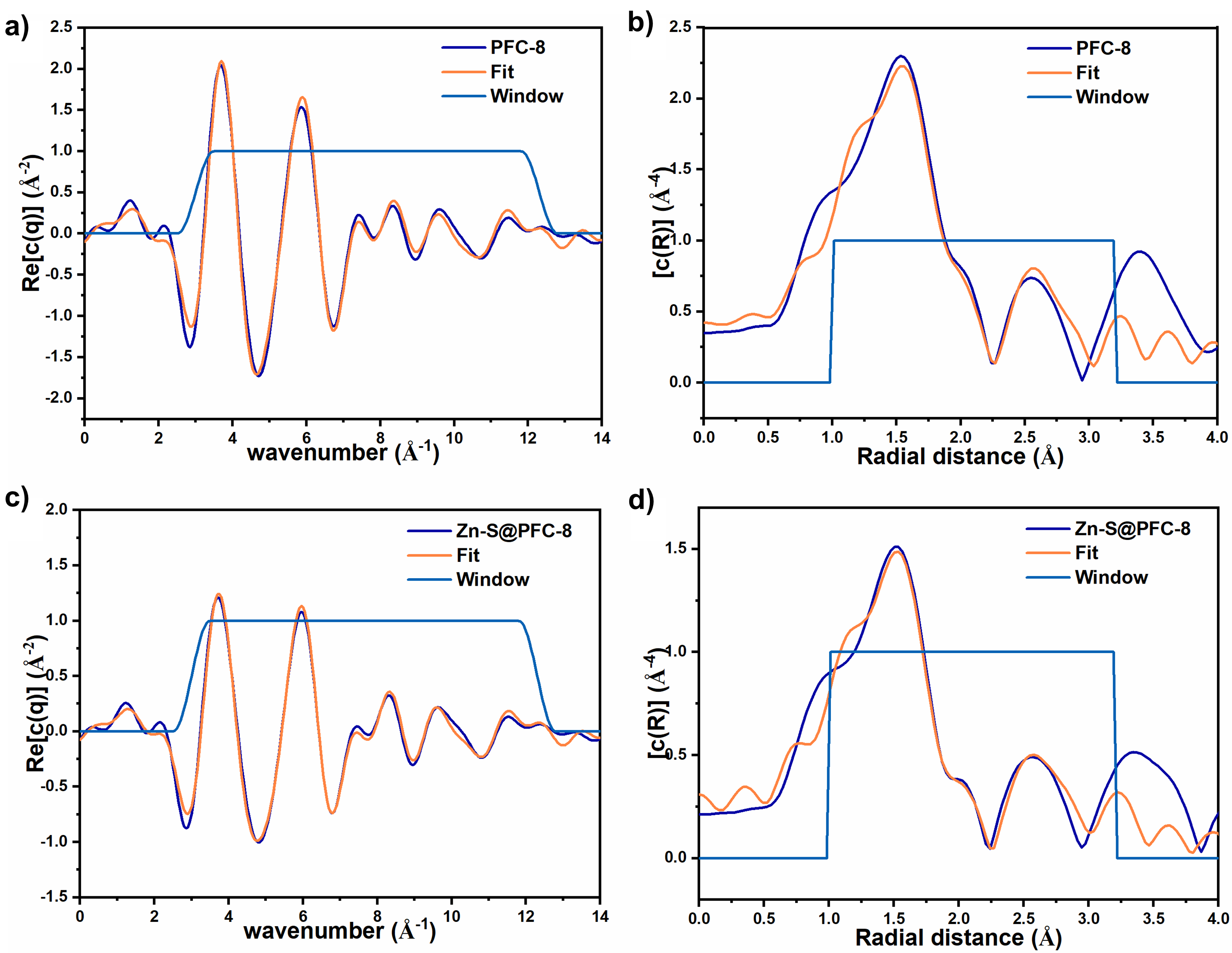


## **Figure S8.** EXAFS fitting curves of **Zn-S@PFC-8** and PFC-8. The blue lines show experimental data, and the yellow lines show the best fitting result, and sky blue lines show the fitting range.


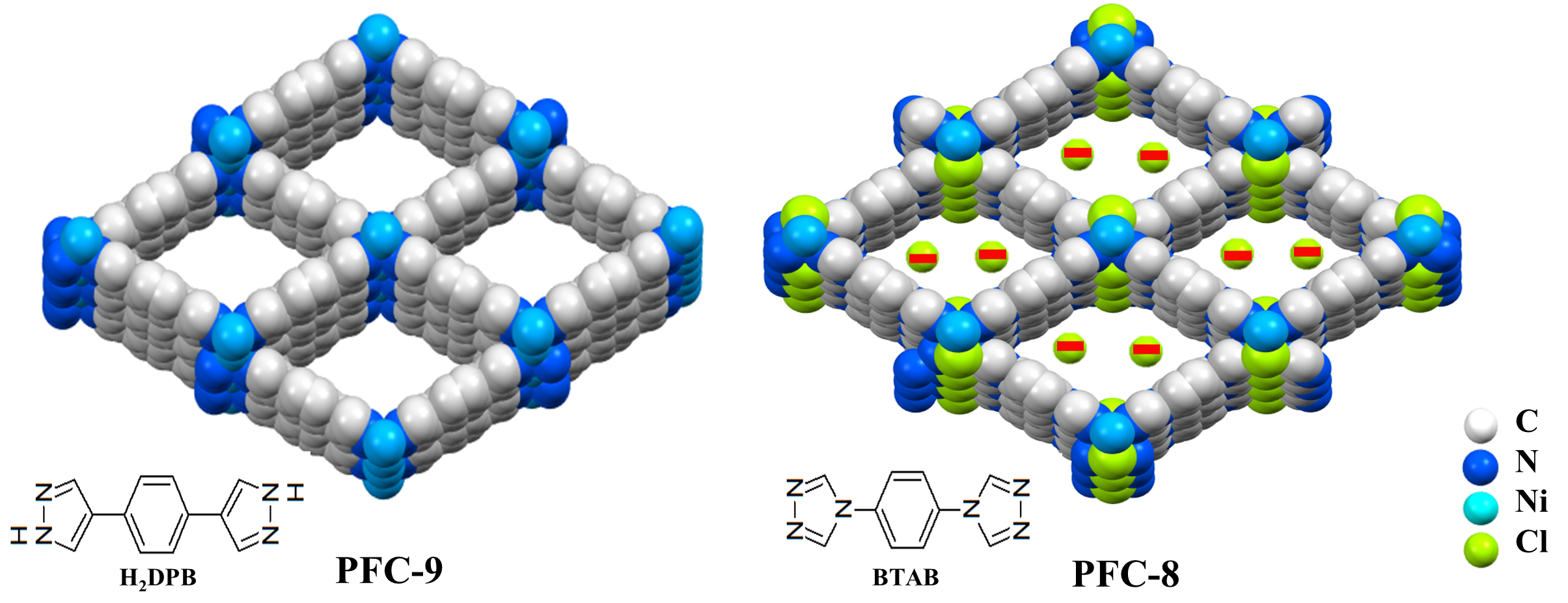


## **Figure S9.** The schematic presentation of the structures of PFC-8 and PFC-9.

## **Table S5.** The AA analyses of **the square shape pellet** samples.

| **Samples** | **Loading (m_1_/m_2_)** |
| --- | --- |
| **PFC-9** | 0% |
| **Zn-S@PFC-9** | 0.06% |

## **Table S6.** The size and conductivity of **the square shape pellet samples** (Length = wide)

| **Samples** | **Thickness (cm)** | **Length (cm)** | **Conductivity (S cm^-1^)** |
| --- | --- | --- | --- |
| **PFC-9** | 0.05 | 0.5 | 7.96×10^-10^ |
| **Zn-S@PFC-9** | 0.05 | 0.5 | 7.20×10^-10^ |


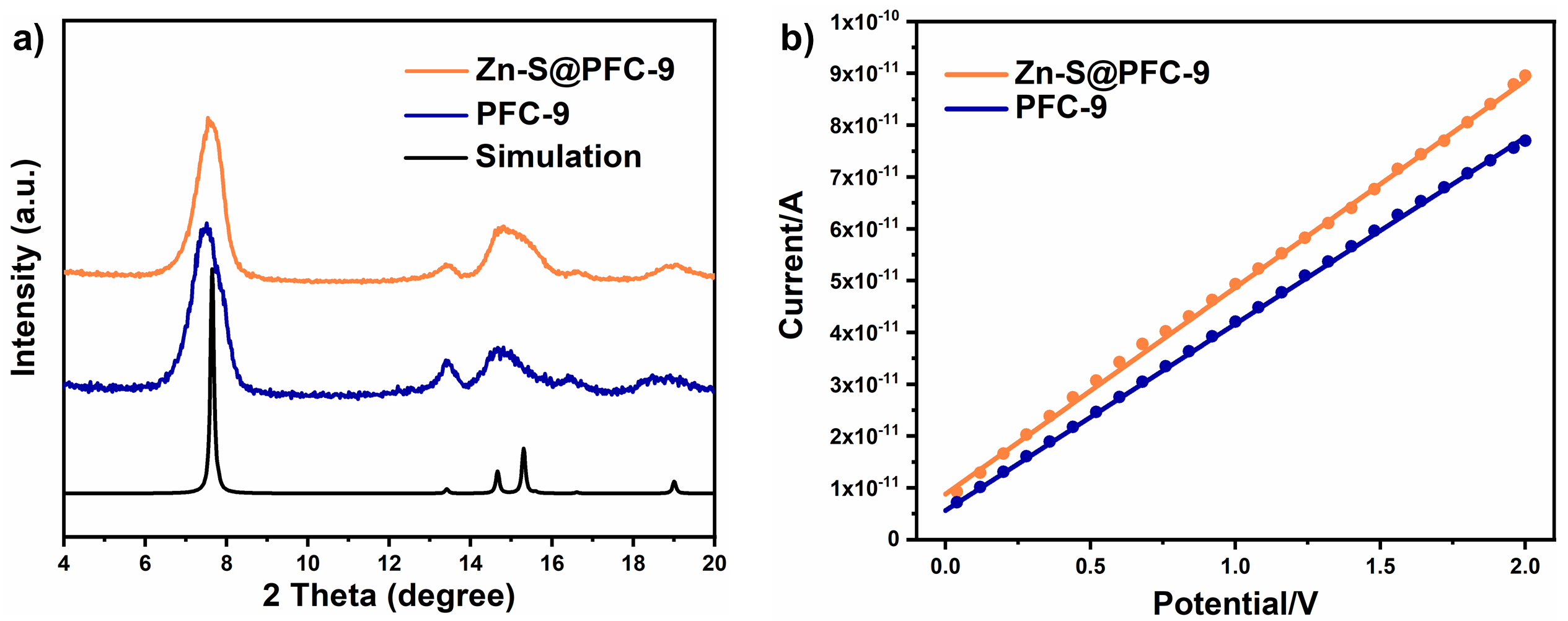


## **Figure S10** a) The PXRD patterns of PFC-9, Zn-S@PFC-9 and PFC-9 simulated. b) The linear fitting of voltage and current of PFC-9, and Zn-S@PFC-9.


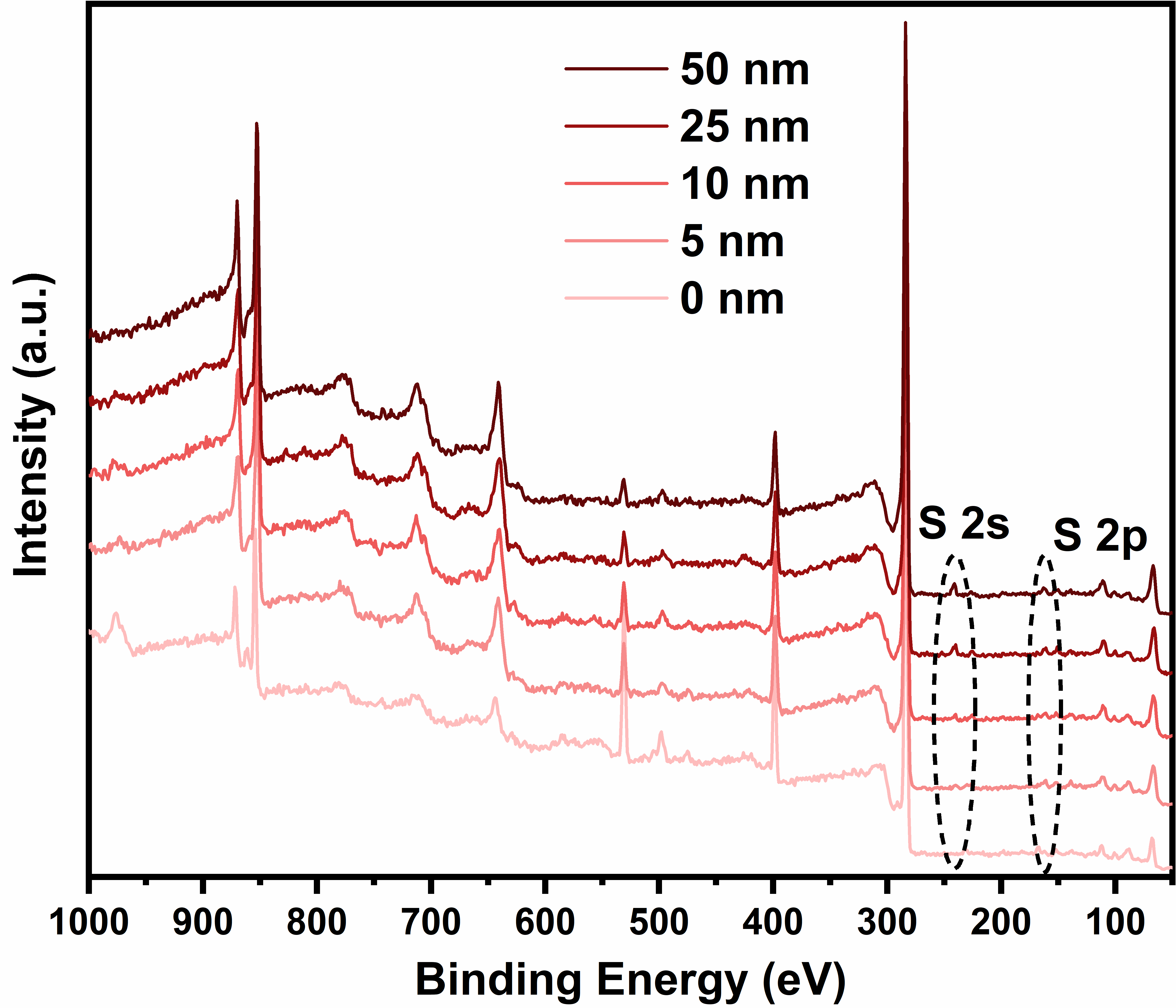


## **Figure S11.** XPS spectra of Zn-S@PFC-9 with different etching depths (0 nm, 5 nm, 10 nm, 25 nm, 50 nm).

In order to further illustrate the inclusion of Zn-S^2-^ inside PFC-9, Zn-S@PFC-9 was also etched with the thickness of 5 nm, 10 nm, 25 nm, 50 nm, respectively, prior to X-ray photoelectron spectroscopy (XPS) analysis. As shown in **Figure S11**, as the depth increases, the intensity of the S element (originated from Zn-S^2-^) gradually appears and stabilizes. We think it may be that the content of Zn-S^2-^ in the PFC-9 is little, so there is no signal on the surface.


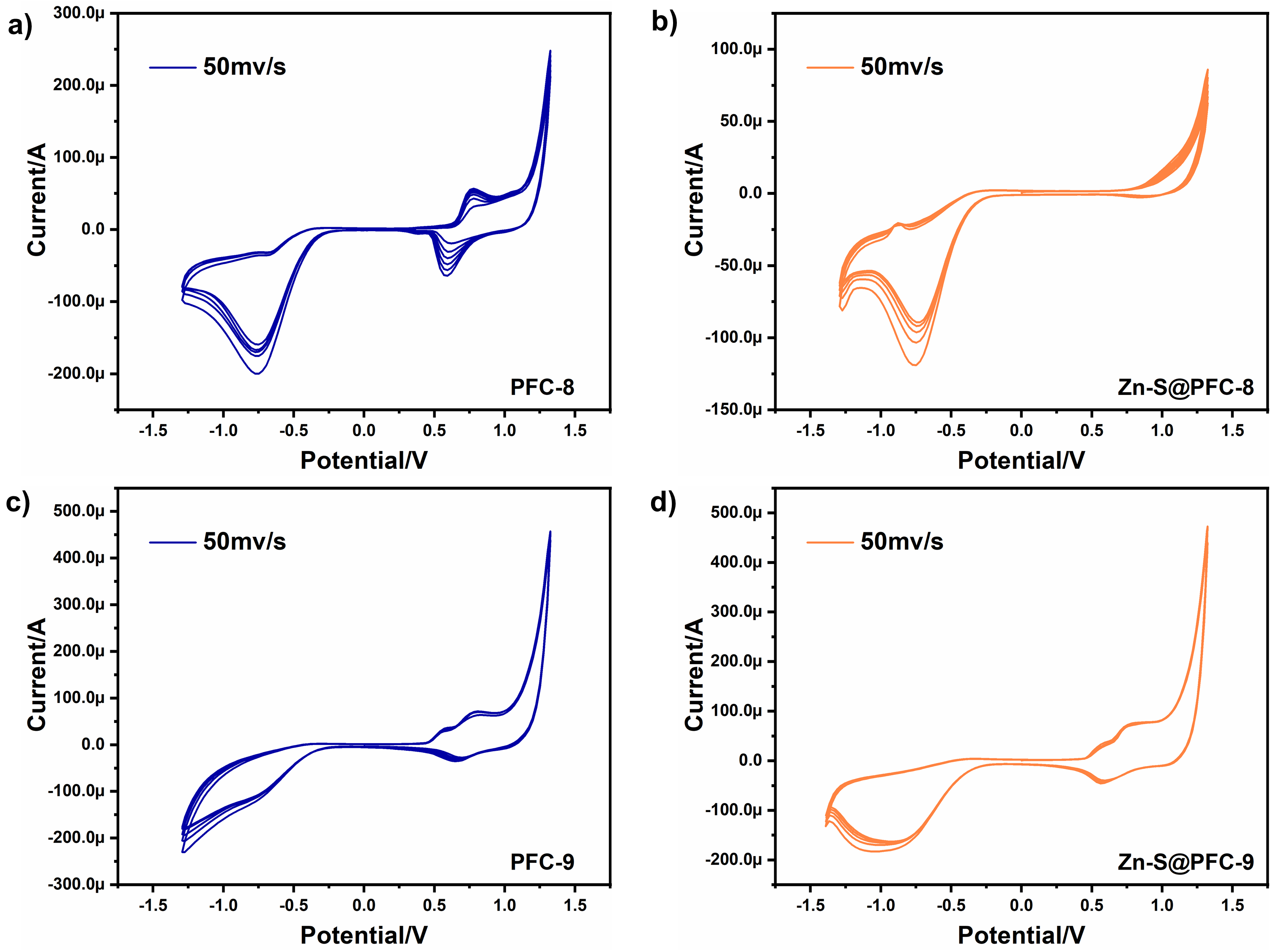


## **Figure S12. a), b)** The cyclic voltammetry of PFC-8, **Zn-S@PFC-8**, **c), d)** PFC-9, and **Zn-S@PFC-9**.

5 mg powdery sample was dispersed in a mixture of 1 mL ethanol and 50 μL 5% Nafion by ultrasonication for several minutes. 100 μL mixture was evenly dropped on a 1×1 cm^2^ window on an ITO glass and dried at room temperature for preparing a working electrode for cyclic voltammogram study. Ag/AgCl electrode and Pt electrode were used as the reference electrode and counter electrode respectively, and 0.2 M Na_2_SO_4_ aqueous solution was used as the electrolyte.

## **Table S7.** Experimentally reported crystal structure of PFC-8. The atom sites with half occupancy are highlighted in red.

| Atom | Wycoff Site | x | y | z | Occupancy |
| --- | --- | --- | --- | --- | --- |
| Ni1 | 4a | 0.5000 | 0.5000 | 0.5000 | 1 |
| Cl1 | 4e | 0.5000 | 0.2500 | 0.6103 | 1 |
| Cl2 | 8i | 0.2881 | 0.2500 | 0.5502 | 0.5 |
| C1 | 16j | 0.3874 | 0.4033 | 0.3847 | 1 |
| C2 | 8i | 0.3019 | 0.2500 | 0.2997 | 1 |
| C3 | 16j | 0.2952 | 0.1208 | 0.2358 | 0.5 |
| C4 | 16j | 0.2570 | 0.3845 | 0.3162 | 0.5 |
| N1 | 16j | 0.4337 | 0.3484 | 0.4318 | 1 |
| N2 | 8i | 0.3569 | 0.2500 | 0.3532 | 1 |
| H1 | 16j | 0.3740 | 0.5430 | 0.3790 | 1 |
| H2 | 16j | 0.3270 | 0.0300 | 0.2250 | 0.5 |
| H3 | 16j | 0.2690 | 0.4980 | 0.3470 | 0.5 |

## **Table S8**. Calculated static total energy of 16 models of PFC-8 considering the orientation of the benzene ring. The mod-6 and mod-11 with the highest space group are highlighted. Note that these two models are essentially the same one and mod-11 is used in the following calculation.

| No. | benzene ring orientation | Space group | Total energy (eV/atom) |
| --- | --- | --- | --- |
| 1 | R – R – R – R | C2/c | -6.2729 |
| 2 | L – R – R – R | P-1 | -6.2730 |
| 3 | R – L – R – R | P-1 | -6.2730 |
| 4 | L – L – R – R | Pnna | -6.2730 |
| 5 | R – R – L – R | P-1 | -6.2730 |
| **6** | **L – R – L – R** | **Pnma** | **-6.2730** |
| 7 | R – L – L – R | C2/m | -6.2730 |
| 8 | L – L – L – R | P-1 | -6.2730 |
| 9 | R – R – R – L | P-1 | -6.2729 |
| 10 | L – R – R – L | C2/m | -6.2729 |
| **11** | **R – L – R – L** | **Pnma** | **-6.2730** |
| 12 | L – L – R – L | P-1 | -6.2730 |
| 13 | R – R – L – L | Pnna | -6.2729 |
| 14 | L – R – L – L | P-1 | -6.2730 |
| 15 | R – L – L – L | P-1 | -6.2730 |
| 16 | L – L – L – L | C2/c | -6.2730 |

## **Table S9.** Calculated total energy and bandgaps for seven models of **Zn-S@PFC-8**.

| Models | Total energy (eV/atom) | Energy gap (eV) |
| --- | --- | --- |
| *a*-mod1 | -6.532 | 0.024 |
| *c*-mod1 | -6.531 | 0.005 |
| *b*-mod1 | -6.531 | - |
| *b*-mod2 | -6.531 | - |
| *b*-mod3 | -6.530 | 0.001 |
| *b*-mod4 | -6.532 | 0.009 |
| *b*-mod5 | -6.532 | 0.054 |


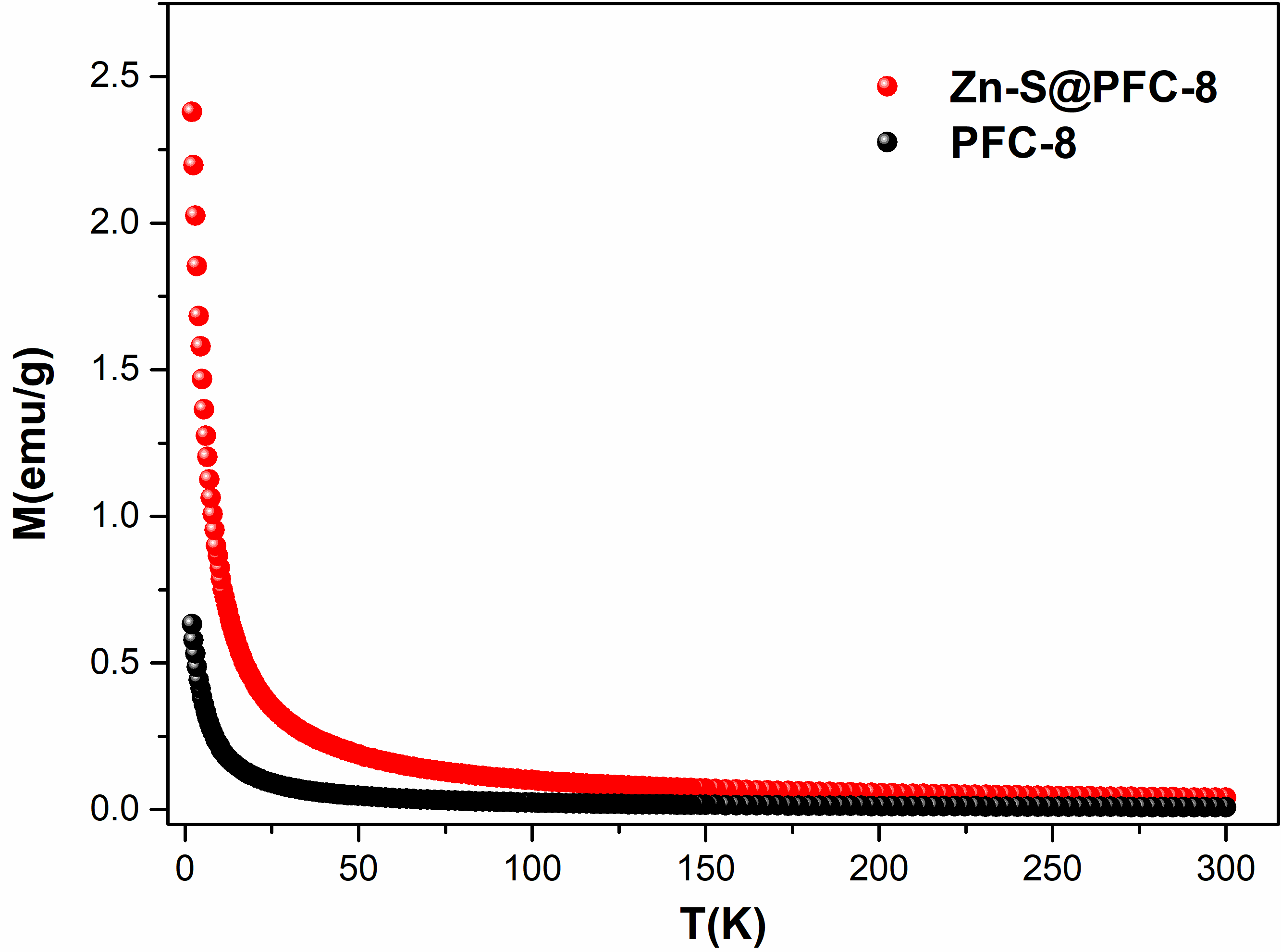


## **Figure S13.** Temperature-dependent magnetization of PFC-8 and **Zn-S@PFC-8**.


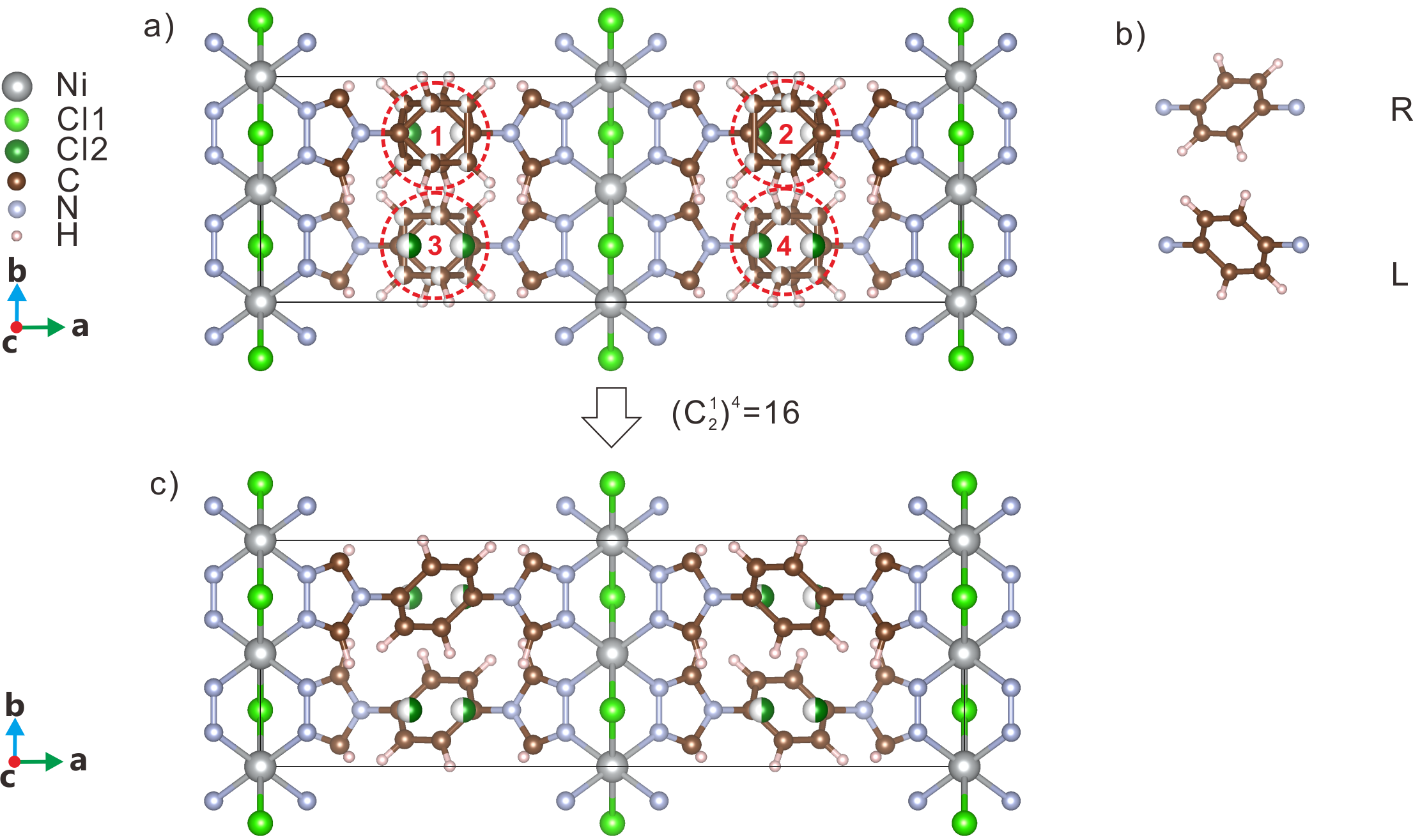


## **Figure S14.** (a) Experimental reported crystal structure of PFC-8. The red dash circles represent the benzene ring with half occupied C and H atoms. (b) Two possible orientation, R and L, for each benzene ring. (c) Structure model of *Pnma* PFC-8.


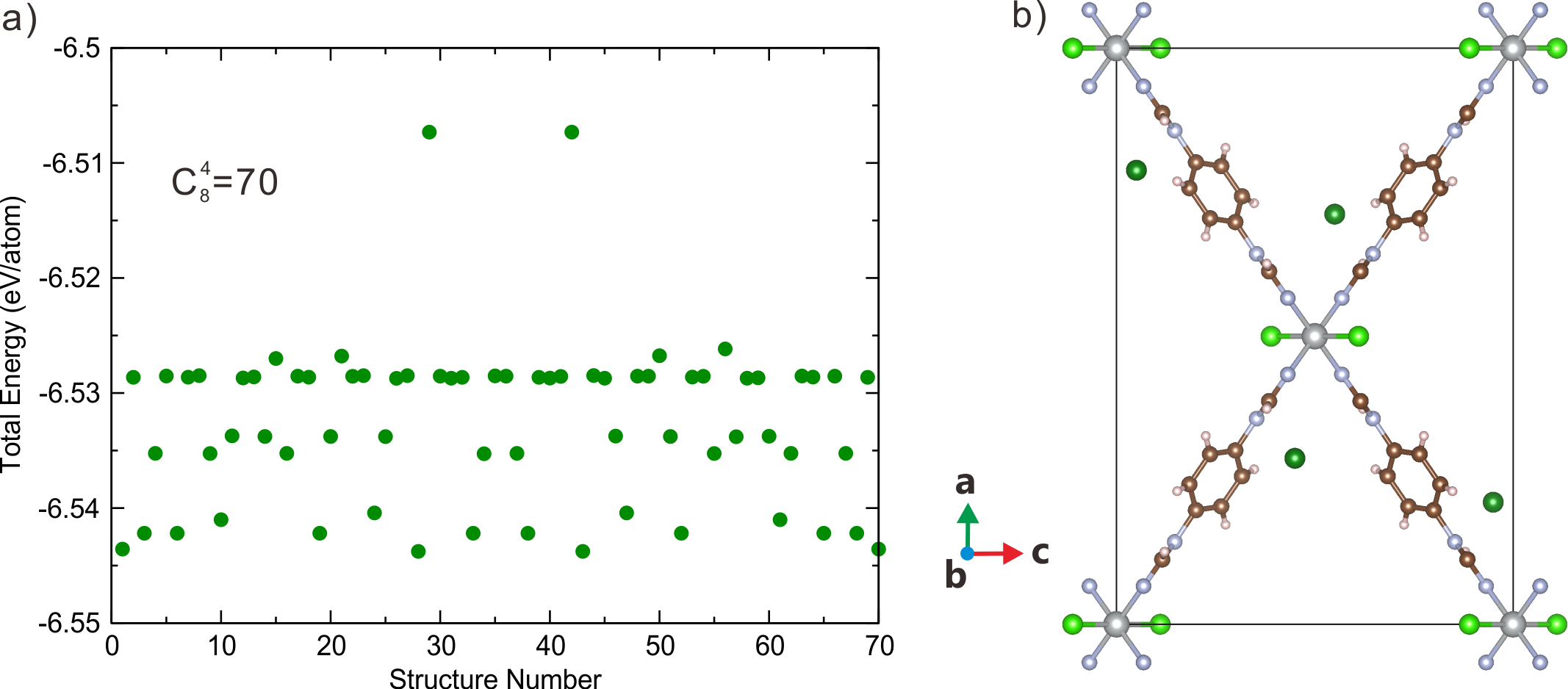


## **Figure S15** The screening of all possible structures due to the half occupied Cl2 atoms. (a) Calculated total energy of 70 structure models. (b) The model with the lowest energy, *P*2_1_2_1_2_1_ PFC-8. Note that, for the convenience of building the models with doped Zn-S^2-^ molecules, the unit cell origin of *P*2_1_2_1_2_1_ PFC-8 are shifted by the vector ***p***=0.5***a***, as presented in **Figure 5a** in the text.


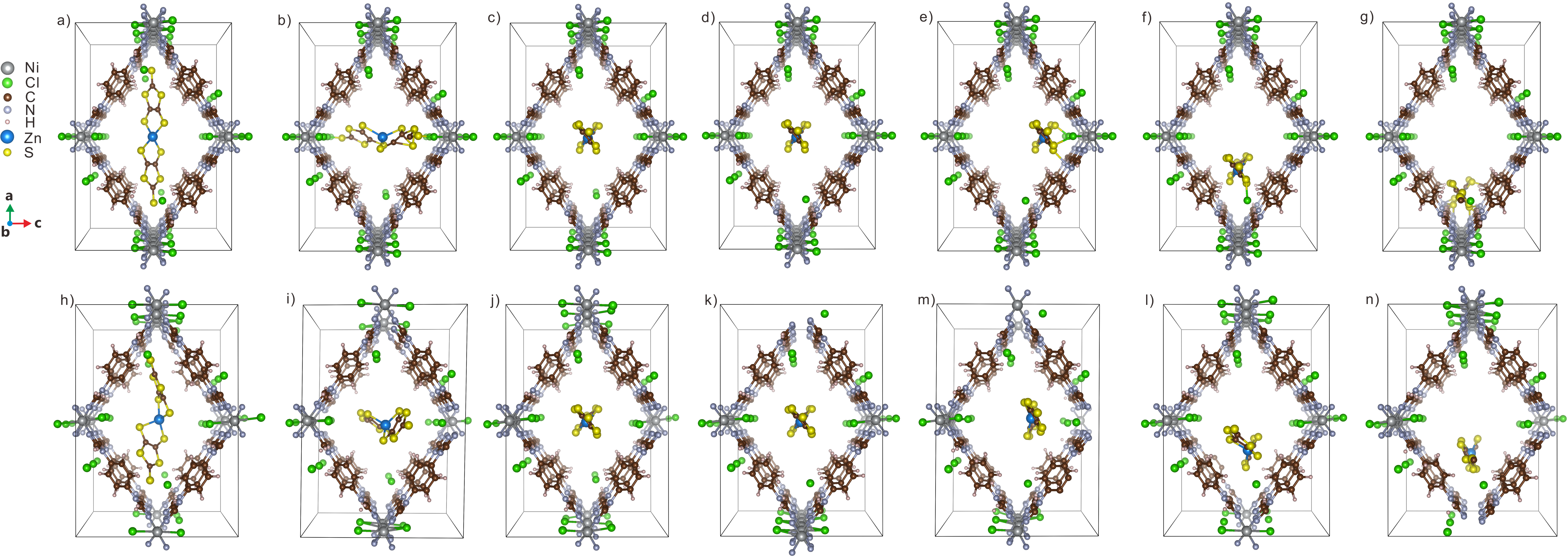


## **Figure S16**. Structure models for **Zn-S@PFC-8**. The initial models (a-g) and the corresponding optimized models (h-n) for *a*-mod1, *c*-mod1, *b*-mod1, *b*-mod2, *b*-mod3, *b*-mod4 and *b*-mod5, respectively. The prefix of these models are named after the orientation of the intercalated Zn-S^2-^ molecule.


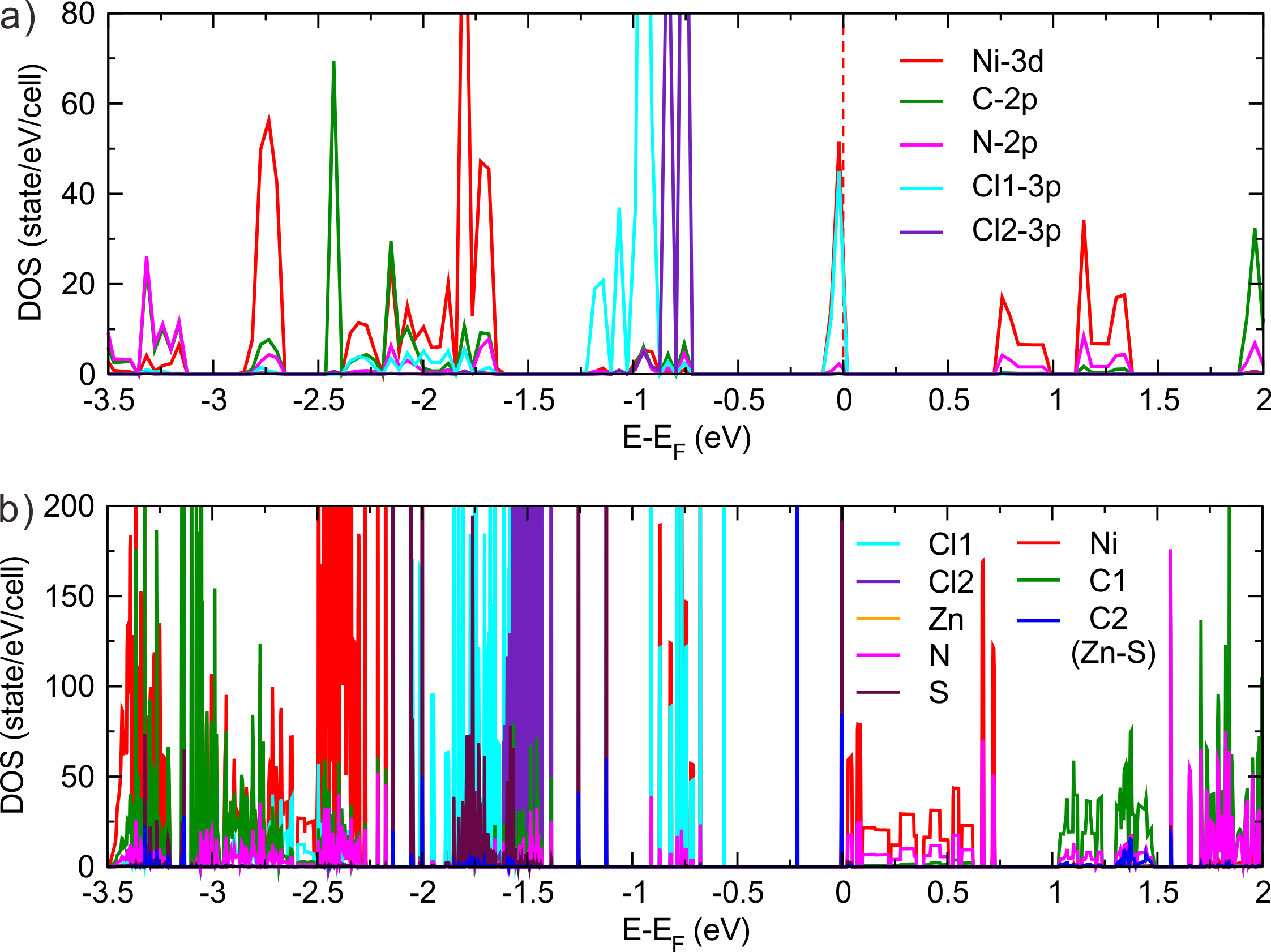


## **Figure S17.** Calculated DOS for (a) *P*2_1_2_1_2_1_ PFC-8 and (b) *a*-mod1 of **Zn-S@PFC-8** at the energy range of -3.5 to 2 eV, The Fermi energy level is set at 0 eV.


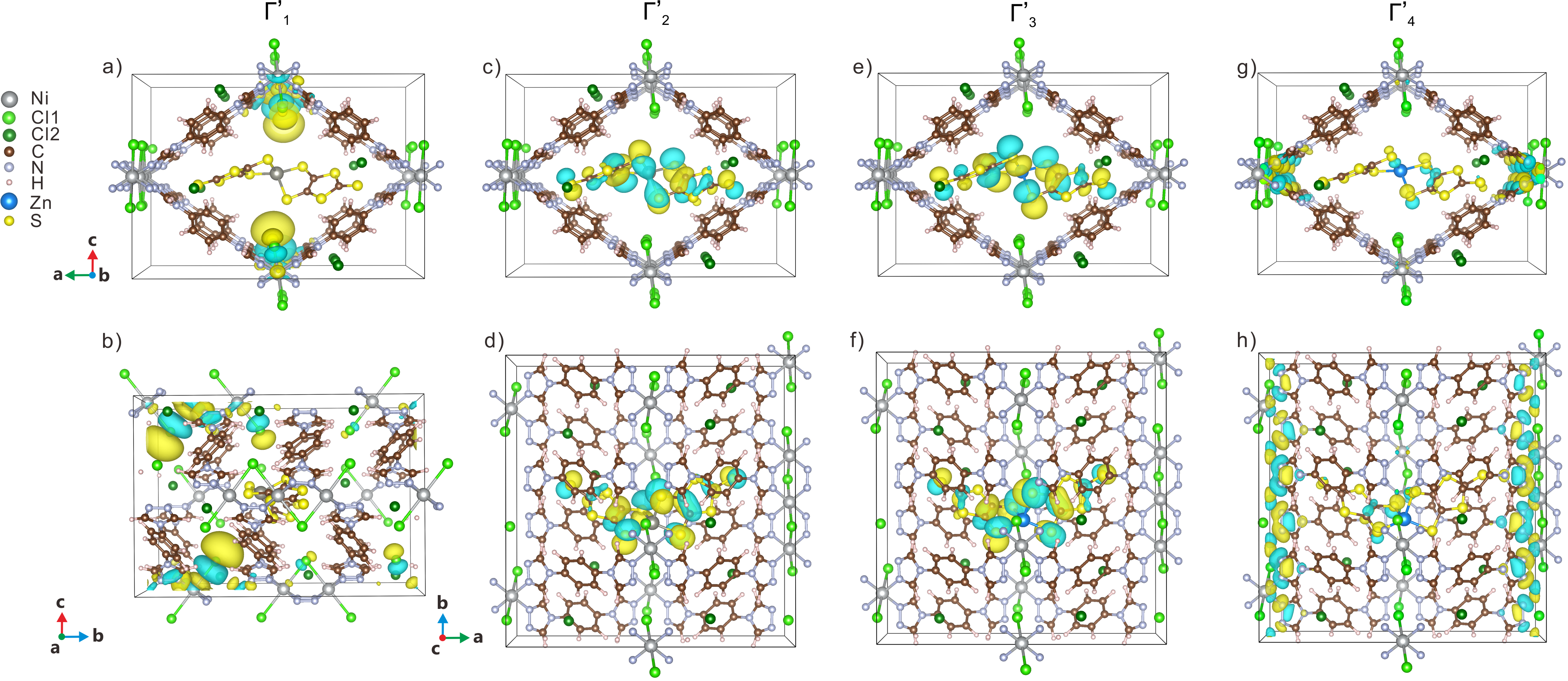


## **Figure S18.** Selected orbitals for a-mod1 Zn-S@PFC-8.

## **Table S10.** Summary of the data for some conductive MOFs.

| **material** | **description, guest** | **σ (S/cm)** | **σ method** | **ref** |
| --- | --- | --- | --- | --- |
| Zn-S@PFC-8 | ^a^Zn-S^2-^ | 1.21×10^-6^ | 2-probe pellet | this work |
|  |  | 1.06×10^-4^ | 2-probe crystal |  |
| Cd(TTF(py)_4_)(TCNQ^•−^)_0.5_(NO_3_) | ^b^TCNQ^•−^ | 2.63 × 10^−8^ | 2-probe pellet | 11 |
| Cd(TTF(py)_4_)(TCNQ^2−^)_0.5_(NO_3_)_0.5_ | ^b^TCNQ^•−^ (air) | 4.77 × 10^−8^ | 2-probe pellet | 11 |
| Cd(TTF(py)_4_)(TCNQ^2−^)_0.5_(NO_3_)_0.5_ | ^b^TCNQ^•−^ (air, I^2-^ treated) | 5.97 × 10^−8^ | 2-probe pellet | 11 |
| Cd(TTF(py)_4_)(TCNQ^•−^)_0.5_(NO_3_)_0.5_ | ^b^I_3_ | 2.16 × 10^−7^ | 2-probe pellet | 11 |
| Cd(TTF(py)_4_)(TCNQ^2−^)_0.5_(NO_3_) | pristine | (1.05 × 10^−8^) | 2-probe pellet | 11 |
| Zn(TTF(py)_4_)(TCNQ^•−^)_0.5_(NO_3_) | ^b^TCNQ^•−^ | 2.48 × 10^−8^ | 2-probe pellet | 11 |
| Cu_2_(TATAB)_3_ | ^b^TCNQ | 2.67 × 10^−7^ (9.75 × 10^−12^) | 2-probe pellet | 12 |
| Cu_3_(BTC)_2_ (HKUST-1) | ^b^TCNQ | 0.07 | 2-probe film | 13 |
|  | ^b^TCNQ | 3 × 10^−3^ | 2-probe pellet | 14 |
|  | ^b^TCNQ | 1.5 × 10^−4^ | 2-probe pellet | 15 |
|  | ^b^TCNQ | 1.92 × 10^−10^ | 2-probe Hg drop film | 16 |
|  | ^b^ferrocene | 3.57 × 10^−15^ | 2-probe Hg drop film | 17 |
|  | pristine | (2 × 10^−9^) | 2-probe film | 18 |
| Fe_3_O(TTFTB)_3_ (MUV-2) | ^c^C_60_ | 4.7 × 10^−9^ (3.7 × 10^−11^) | 2-probe pellet | 19 |
| Zn(TPP) | ^c^C_60_ | 1.5 × 10^−11^ (2 × 10^−13^) | 2-probe film | 20 |
| Zr_6_(OH)_16_(TBAPy)_2_(NU-1000) | ^b^Ni(IV) bis(dicarbollide) | 4.3 × 10^−9^ (9.1 × 10^−12^) | 2-probe pellet | 21 |
| Zr_6_(OH)_16_(TBAPy)_2_(NU-901) | ^c^C_60_ | 1 × 10^−3^ (<10^−14^) | 2-probe pellet | 22 |
| Mn_2_(TTFTB) |  | 8.6 × 10^-5^ | 2-probe crystal | 23 |
| Co_2_(TTFTB) |  | 1.5× 10^-5^ | 2-probe crystal | 23 |
| Cd_2_(TTFTB) |  | 2.9× 10^-4^ | 2-probe crystal | 23 |
| NNU-27 |  | 1.3× 10^-3^ | 2-probe crystal | 24 |
| Zn_2_(TTFTB) |  | 4.0× 10^-6^ | 2-probe crystal | 25 |
| Tb(Cu_4_I_4_)(PCA)_3_ | ^b^I_2_ | 2.16 × 10^−4^ | 2-probe crystal | 26 |
| Zn_3_(lac)_2_(pybz)_2_ | ^b^I_2_ | 1.65 × 10^−4^ | 2-probe crystal | 27 |

a. Anionic conductive organic molecules (Electrostatic interaction), b. Neutral conductive organic molecules (Van der Waals interaction), c. Conductive inorganic molecules (Van der Waals interaction).

## **Reference**

(1) Hohenberg, P.; Kohn, W., Inhomogeneous Electron Gas. *Phys. Rev.* **1964,** *136*, B864-B871.

(2) Kohn, W.; Sham, L. J., Self-Consistent Equations Including Exchange and Correlation Effects. *Phys. Rev.* **1965,** *140*, A1133-A1138.

(3) Kresse, G.; Hafner, J., Ab initio molecular dynamics for liquid metals. *Phys. Rev. B Condens. Matter.* **1993,** *47*, 558-561.

(4) Kresse, G.; Furthmuller, J., Efficiency of ab-initio total energy calculations for metals and semiconductors using a plane-wave basis set. *Comp. Mater. Sci.* **1996,** *6*, 15-50.

(5) Kresse, G.; Furthmuller, J., Efficient iterative schemes for ab initio total-energy calculations using a plane-wave basis set. *Phys. Rev. B* **1996,** *54*, 11169-11186.

(6) Blochl, P. E., Projector augmented-wave method. *Phys. Rev. B Condens. Matter.* **1994,** *50*, 17953-17979.

(7) Perdew, J. P.; Burke, K.; Ernzerhof, M., Generalized gradient approximation made simple. *Phys. Rev. Lett.* **1996,** *77*, 3865-3868.

(8) Perdew, J. P.; Wang, Y., Accurate and simple analytic representation of the electron-gas correlation energy. *Phys. Rev. B Condens. Matter.* **1992,** *45*, 13244-13249.

(9) Anisimov, V. I.; Poteryaev, A. I.; Korotin, M. A.; Anokhin, A. O.; Kotliar, G., First-principles calculations of the electronic structure and spectra of strongly correlated systems: dynamical mean-field theory. *J. Phys-Condens. Mat.* **1997,** *9*, 7359-7367.

(10) Dudarev, S. L.; Botton, G. A.; Savrasov, S. Y.; Humphreys, C. J.; Sutton, A. P., Electron-energy-loss spectra and the structural stability of nickel oxide: An LSDA+U study. *Phys. Rev. B* **1998,** *57*, 1505-1509.

(11) Wang, H. Y.; Su, J.; Ma, J. P.; Yu, F.; Leong, C. F.; D'Alessandro, D. M.; Kurmoo, M.; Zuo, J. L., Concomitant Use of Tetrathiafulvalene and 7,7,8,8-Tetracyanoquinodimethane within the Skeletons of Metal-Organic Frameworks: Structures, Magnetism, and Electrochemistry. *Inorg. Chem.* **2019**, 58, 8657-8664.

(12) Huang, Q.-Q.; Lin, Y.-J.; Zheng, R.; Deng, W.-H.; Kashi, C.; Kumar, P. N.; Wang, G.-E.; Xu, G., Tunable electrical conductivity of a new 3D MOFs: Cu-TATAB. *Inorg. Chem. Commun.* **2019,** *105*, 119-124.

(13) Talin, A. A.; Centrone, A.; Ford, A. C.; Foster, M. E.; Stavila, V.; Haney, P.; Kinney, R. A.; Szalai, V.; El Gabaly, F.; Yoon, H. P.; Leonard, F.; Allendorf, M. D., Tunable electrical conductivity in metal-organic framework thin-film devices. *Science* **2014,** *343*, 66-69.

(14) Chen, X.; Wang, Z.; Hassan, Z. M.; Lin, P.; Zhang, K.; Baumgart, H.; Redel, E., Seebeck Coefficient Measurements of Polycrystalline and Highly Ordered Metal-Organic Framework Thin Films. *ECS J. Solid State Sci. and Technol.* **2017,** *6*, P150-P153.

(15) Schneider, C.; Ukaj, D.; Koerver, R.; Talin, A. A.; Kieslich, G.; Pujari, S. P.; Zuilhof, H.; Janek, J.; Allendorf, M. D.; Fischer, R. A., High electrical conductivity and high porosity in a Guest@MOF material: evidence of TCNQ ordering within Cu_3_BTC_2_ micropores. *Chem. Sci.* **2018,** *9*, 7405-7412.

(16) Neumann, T.; Liu, J.; Wachter, T.; Friederich, P.; Symalla, F.; Welle, A.; Mugnaini, V.; Meded, V.; Zharnikov, M.; Woll, C.; Wenzel, W., Superexchange Charge Transport in Loaded Metal Organic Frameworks. *ACS Nano* **2016,** *10*, 7085-7093.

(17) Liu, J.; Wachter, T.; Irmler, A.; Weidler, P. G.; Gliemann, H.; Pauly, F.; Mugnaini, V.; Zharnikov, M.; Woll, C., Electric transport properties of surface-anchored metal-organic frameworks and the effect of ferrocene loading. *ACS Appl. Mater. Interfaces* **2015,** *7*, 9824-9830.

(18) Dolgopolova, E. A.; Brandt, A. J.; Ejegbavwo, O. A.; Duke, A. S.; Maddumapatabandi, T. D.; Galhenage, R. P.; Larson, B. W.; Reid, O. G.; Ammal, S. C.; Heyden, A.; Chandrashekhar, M.; Stavila, V.; Chen, D. A.; Shustova, N. B., Electronic Properties of Bimetallic Metal-Organic Frameworks (MOFs): Tailoring the Density of Electronic States through MOF Modularity. *J. Am. Chem. Soc.* **2017,** *139*, 5201-5209.

(19) Souto, M.; Calbo, J.; Manas-Valero, S.; Walsh, A.; Minguez Espallargas, G., Charge-transfer interactions between fullerenes and a mesoporous tetrathiafulvalene-based metal-organic framework. *Beilstein J. Nanotechnol.* **2019,** *10*, 1883-1893.

(20) Liu, X.; Kozlowska, M.; Okkali, T.; Wagner, D.; Higashino, T.; Brenner-Weiss, G.; Marschner, S. M.; Fu, Z.; Zhang, Q.; Imahori, H.; Brase, S.; Wenzel, W.; Woll, C.; Heinke, L., Photoconductivity in Metal-Organic Framework (MOF) Thin Films. *Angew. Chem. Int. Ed.* **2019,** *58*, 9590-9595.

(21) Kung, C. W.; Otake, K.; Buru, C. T.; Goswami, S.; Cui, Y.; Hupp, J. T.; Spokoyny, A. M.; Farha, O. K., Increased Electrical Conductivity in a Mesoporous Metal-Organic Framework Featuring Metallacarboranes Guests. *J. Am. Chem. Soc.* **2018,** *140*, 3871-3875.

(22) Goswami, S.; Ray, D.; Otake, K. I.; Kung, C. W.; Garibay, S. J.; Islamoglu, T.; Atilgan, A.; Cui, Y.; Cramer, C. J.; Farha, O. K.; Hupp, J. T., A porous, electrically conductive hexa-zirconium(iv) metal-organic framework. *Chem. Sci.* **2018,** *9*, 4477-4482.

(23) Sun, L.; Hendon, C. H.; Minier, M. A.; Walsh, A.; Dinca, M., Million-Fold Electrical Conductivity Enhancement in Fe_2_(DEBDC) versus Mn_2_(DEBDC) (E = S, O). *J. Am. Chem. Soc.* **2015,** *137*, 6164-6167.

(24) Chen, D.; Xing, H.; Su, Z.; Wang, C., Electrical conductivity and electroluminescence of a new anthracene-based metal-organic framework with pi-conjugated zigzag chains. *Chem. Commun. (Camb)* **2016,** *52*, 2019-2022.

(25) Narayan, T. C.; Miyakai, T.; Seki, S.; Dinca, M., High charge mobility in a tetrathiafulvalene-based microporous metal-organic framework. *J. Am. Chem. Soc.* **2012,** *134*, 12932-12935.

(26) Hu, Y. Q.; Li, M. Q.; Wang, Y.; Zhang, T.; Liao, P. Q.; Zheng, Z.; Chen, X. M.; Zheng, Y. Z., Direct Observation of Confined I^-…^I_2_…I^-^ Interactions in a Metal-Organic Framework: Iodine Capture and Sensing. *Chemistry* **2017,** *23*, 8409-8413.

(27) Zeng, M. H.; Wang, Q. X.; Tan, Y. X.; Hu, S.; Zhao, H. X.; Long, L. S.; Kurmoo, M., Rigid pillars and double walls in a porous metal-organic framework: single-crystal to single-crystal, controlled uptake and release of iodine and electrical conductivity. *J. Am. Chem. Soc.* **2010,** *132*, 2561-2563.
